# Surface Coatings on Biomedical Magnesium Alloys

**DOI:** 10.3390/ma18143411

**Published:** 2025-07-21

**Authors:** Jiapeng Ren, Zhenyu Zhao, Hua Li, Dongsheng Wang, Cijun Shuai, Youwen Yang

**Affiliations:** 1 Intelligent Manufacturing and Equipment School, Shenzhen Institute of Information Technology, Shenzhen 518000, China; 6720230614@mail.jxust.edu.cn (J.R.);; 2 School of Mechanical and Electrical Engineering, Jiangxi University of Science and Technology, Ganzhou 341000, China; 3 School of Mechanical Engineering, Tongling University, Tongling 244000, China

**Keywords:** magnesium alloys, biodegradable materials, corrosion, surface modification, coatings

## Abstract

Magnesium (Mg) alloys have demonstrated tremendous potential in biomedical applications, emerging as promising metallic biomaterials due to their biocompatibility, degradability, and favorable mechanical properties. However, their practical implementation faces significant limitations stemming from mechanical performance degradation and premature fracture failure caused by complex physiological interactions, including flow erosion, corrosion fatigue, stress coupling effects, and dynamic wear under bodily conditions. Surface coating technology has been recognized as an effective strategy to prevent direct contact between magnesium substrates and corrosive media. This review systematically examines the fundamental degradation mechanisms of magnesium alloys in both vivo and vitro environments, presents recent advances in surface modification coatings for magnesium alloys, and critically analyses the interaction mechanisms between modified layers and electrolyte solutions. Special emphasis is placed on revealing the formation mechanisms, structural characteristics, and fracture behaviors of conversion coatings. Furthermore, the study discusses the current challenges in biomedical surface modification of magnesium alloys, proposes potential solutions to enhance their clinical applicability, and outlines future research directions to fully exploit the development potential of these advanced biomaterials.

## 1. Introduction

Magnesium alloys have garnered extensive attention in the development of biomedical products, such as orthopedic fixation devices and cardiovascular stents, in recent years, owing to their unique combination of mechanical properties, biocompatibility, and biodegradability [1]. Pure magnesium exhibits a density of 1.74 g/cm^3^, nearly identical to that of human bone (1.75 g/cm^3^), while its elastic modulus (45 GPa) closely approximates that of cortical bone (15–30 GPa) [2]. The superior matching of yield strength with natural bone tissue enables magnesium alloys to effectively mitigate stress-shielding effects caused by elastic modulus mismatch [3], which proves clinically significant for promoting fracture healing and extending implant service life. Compared to other metallic materials, magnesium’s higher chemical reactivity prevents its free existence in nature, rendering it prone to aqueous corrosion; it is particularly vulnerable in chloride-rich physiological environments containing Cl^−^ ions [4]. This inherent corrosion susceptibility has been strategically utilized in medical applications to develop magnesium-based alloys as biodegradable hard tissue implants, which gradually degrade while fulfilling their functions and are eventually absorbed or excreted by the human body. Magnesium alloys demonstrate superior biocompatibility compared to other biomedical metals, particularly considering that magnesium serves as an essential nutritional element with a recommended daily intake (240–420 mg/day) significantly higher than that of zinc and iron [5]. Unlike iron degradation products, magnesium’s corrosion byproducts can be effectively assimilated or eliminated through physiological processes, thereby reducing complications from secondary removal surgeries and associated healthcare burdens [6].

Although magnesium has the above excellent performance characteristics, its corrosion resistance is still an important factor limiting its wide use. The reason for its poor corrosion resistance is mainly because the chemical properties of magnesium are relatively active and easily react chemically with water molecules in the air to generate MgO and Mg(OH)_2_-dominated films on the surface, which have poor mechanical properties. The structure is relatively loose, so it cannot continue to protect the inner layer of magnesium alloy [7], and because the corrosion potential of magnesium is the lowest among all metals, only −2.37 V, galvanic corrosion easily develops with other metals as the anode material [8]. Due to its poor corrosion resistance, magnesium exhibits a faster corrosion rate in the human body, which is accompanied by premature loss of mechanical properties, resulting in premature failure of implants and affecting tissue healing [9].

Current studies have shown that the mechanical stability of most magnesium alloy implants in the physiological environment can only be maintained for 6–8 weeks, while hard tissue repair materials that can be used for clinical degradation should be stable for at least 12 weeks after implantation in the human body, so that tissues have sufficient time for growth and healing [1,10]. Therefore, improving the corrosion resistance of magnesium alloy surfaces is a prerequisite to ensure good application results in this field [11].

To address the issue of excessively rapid degradation caused by corrosion, two predominant approaches are currently employed: surface modification and bulk material optimization [12,13]. The bulk optimization methodology is further categorized into three principal strategies, as follows:

(1) Material Alloying [14]: By designing appropriate alloying strategies involving elements such as Fe [15], Al [16], Ca [17], Zn [18], Zr [19], and Y [20], the mechanisms of grain boundary strengthening and solid solution hardening can be achieved, which effectively reduces the corrosion rate of magnesium to some extent. Gu et al. [21] fabricated Sr-Mg binary alloys with varying Sr contents through alloying and hot-rolling processes, demonstrating that the addition of 0–2 wt% Sr reduced micro-shrinkage porosity and refined grain size, consequently enhancing both corrosion resistance and corrosion uniformity. However, excessive Sr addition resulted in deteriorated mechanical properties and accelerated corrosion rates in the as-rolled Mg-Sr alloys. Although alloying remains widely employed, significant challenges persist in corrosion protection, as these alloys lack the capacity to form protective oxide layers on their surfaces, leading to uncontrolled corrosion rates [10]. Moreover, extensive alloying may induce galvanic coupling reactions in Mg alloys, thereby reducing the corrosion resistance of the Mg matrix. The majority of alloying elements tend to form detrimental impurities or secondary phases that adversely affect the microstructure [22].
(2) Amorphous Phase Engineering [23]: Metallic glasses exhibit unique structural characteristics including single-phase homogeneous solid solutions and multi-component compositions. This chemically homogeneous structure eliminates intergranular corrosion but increases susceptibility to localized pitting and selective corrosion in amorphous alloys. The inevitable stress exposure during implantation makes amorphous alloys particularly vulnerable to corrosion fatigue and stress corrosion cracking, which significantly accelerates degradation and reduces service lifespan [24]. Zhou et al. [25] synthesized Mg_68−x_Zn_28_Ca_4_Nd_x_ (x = 0, 0.5, 1, 1.5) alloys with 2 mm diameters to investigate the effects of Nd content on glass-forming ability and corrosion resistance. The Mg_67.5_Zn_28_Ca_4_Nd_0.5_ amorphous specimen demonstrated optimal corrosion resistance among the tested compositions. Li et al. [26] investigated the corrosion fatigue behavior of Mg_66_Zn_30_Ca_3_Sr_1_ amorphous alloy in phosphate-buffered saline under cyclic loading conditions. Their findings revealed that cyclic loading accelerates corrosion rates through repeated elastic deformation that disrupts passive films, inducing galvanic corrosion and crevice corrosion, ultimately leading to localized exfoliation and catastrophic brittle fractures in amorphous alloys. The study concluded that fatigue corrosion in amorphous alloys exhibits high sensitivity to cyclic stress levels.
(3) Processing Optimization: Techniques such as extrusion and rolling can refine the grain structure of Mg alloys, thereby enhancing their corrosion resistance. Cai et al. [27] melted Mg–Zn alloy materials in an electric resistance furnace at 750–800 °C, followed by casting into permanent steel molds preheated to 200 °C, ultimately fabricating Mg–5Zn alloy specimens with a measured tensile strength of 194.59 MPa and self-corrosion potential of −1.477 V. Jana et al. [28] prepared pure magnesium and Mg–Gd–Nd–Zr–Zn alloys through powder compaction sintering with subsequent heat treatment, demonstrating that post-treatment materials exhibited superior compressive strength compared to as-sintered counterparts. The heat-treated magnesium alloy displayed a self-corrosion potential of −1.49 V, whereas the untreated material registered −1.51 V, indicating improved electrochemical stability through thermal processing. Although processing optimization can enhance both corrosion resistance and mechanical properties, certain manufacturing techniques still fail to meet clinically required mechanical performance standards and biocompatibility thresholds.

Surface modification has emerged as a prominent research focus in recent years, demonstrating significant potential for effectively enhancing the performance of biomedical magnesium alloys [29,30,31]. This review systematically examines the degradation mechanisms of magnesium alloys in both in vivo and in vitro environments from the perspective of corrosion fundamentals. The interaction mechanisms between modified surface layers and electrolyte solutions are critically analyzed, with particular emphasis on the failure mechanisms of coating layers in biological media. Building upon fundamental corrosion mechanisms, the control strategies and recent advancements in surface coating technologies for magnesium alloys are comprehensively discussed. State-of-the-art coating techniques including conversion coatings, hydrothermal coatings, and layer-by-layer coatings are comparatively reviewed, with detailed analysis of their respective technical merits and limitations. Finally, this work identifies existing challenges in biomedical magnesium alloy surface modification and proposes strategic directions for future research development. The representative studies of various coatings applied to Mg alloys are shown in Table 1.

## 2. Degradation Mechanism of Magnesium Alloy

The chemical properties of magnesium alloy are extremely active and will react with oxygen to form an oxide film in the exposed air. The oxide film formed in the air is relatively loose and its density coefficient *α* is only 0.779, which has a very limited protective effect on the magnesium matrix [10]. In a complex and highly corrosive human environment, its corrosion mechanism is also very complex. During the degradation of magnesium alloy, hydroxide and hydrogen are produced with the aqueous solution. The tendency of magnesium alloy to be corroded will increase, and the chemical equation of the reaction is shown as follows:

(1) $$\begin{array}{c} Mg\to {Mg}^{2+}+2e^{-} \end{array}$$

(2) $$\begin{array}{c} 2H_{2}O+2e^{-}\to 2OH^{-}+H_{2}↑ \end{array}$$

(3) $$\begin{array}{c} Mg+2H_{2}O\to Mg{\left(OH\right)}_{2}+H_{2}↑ \end{array}$$

The corrosion behavior of magnesium alloy is closely related to the pH value of the solution. The literature [3] report that the stable pH ranges of MgO and Mg(OH)_2_ are pH ≥ 13.83 and pH ≥ 11.46, respectively. Therefore, when the pH of the surrounding environment is lower than 11.46, both MgO and Mg(OH)_2_ will dissolve, resulting in increased corrosion of magnesium, and the corrosion rate will gradually increase with the decrease of pH. Therefore, in a neutral fluid environment with a pH of about 7.4, the corrosion resistance of magnesium will become extremely poor. The human physiological environment contains a large number of aggressive ions such as Cl^−^, HPO_4_^2−^, SO_4_^2−^, etc. The corrosion rate of magnesium alloy in this acidic solution is very high. Especially when the concentration of Cl^−^ is high, it has a strong corrosion damage effect on the surface of magnesium alloy, and its reaction formula is as follows:

(4) $$\begin{array}{c} Mg{\left(OH\right)}_{2}+2{Cl}^{-}\to M{gCl}_{2}+2{OH}^{-} \end{array}$$

**Figure 1:**
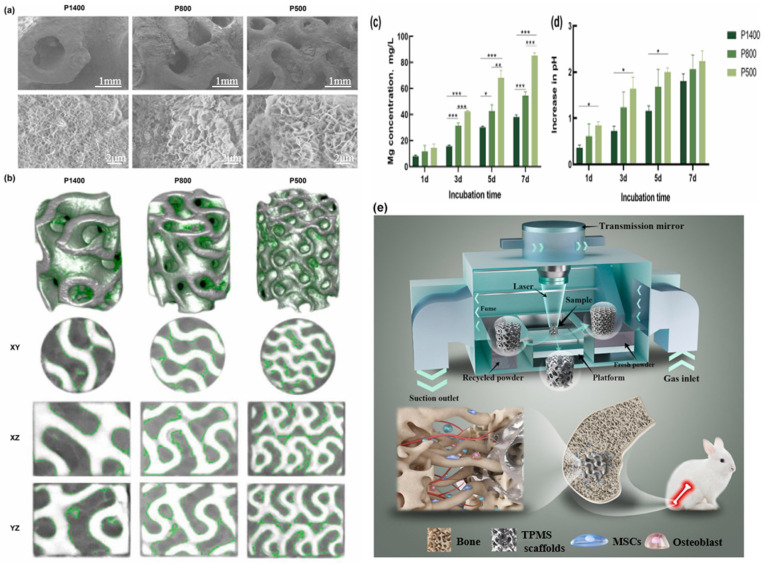
(**a**) SEM images; (**b**) Micro-CT images of the degradation products of porous magnesium scaffolds with different pore sizes; (**c**) Magnesium ion release from the porous scaffolds with different pore sizes; and (**d**) Changes in pH value. (**e**) Schematic diagram illustrating the implantation of the magnesium alloy in vivo. The statistical significance was indicated by * *p* < 0.05, ** *p* < 0.01 and *** *p* < 0.001 [63].

The solubility of Cl^−^ is large, the radius is small, and it easily penetrates the surface film when in contact with the matrix, which can promote the diffusion of the cation and increase the corrosion current. At the same time, the Mg(OH)_2_/MgO corrosion products generated on the surface are converted into MgCl_2_, which is easily soluble in water. The dissolution of Mg(OH)_2_ or MgO will weaken the protective effect of the corrosion products, increase the surface’s active area, accelerate the corrosion of the matrix, and lead to pitting on the surface, which will fail before the tissue is healed. The fixed and supporting effect on the injured part is lost, which affects the growth and healing of the injured tissue [62]. In addition, amino acids, proteins, and various sugars in the physiological environment can accelerate the degradation of magnesium alloys. As shown in Figure 1, Wang [63] analyzed the in vitro degradation behavior of the magnesium scaffold. Figure 1a displays the morphology of the degradation products, for which EDS measurements were performed. The degradation products on the scaffold surface were composed of Mg(OH)_2_, with surface enrichment of calcium and phosphorus observed, indicating the deposition of calcium phosphate. Figure 1c,d present the magnesium ion release and pH values of the scaffold at different time points. Both the magnesium ion concentration and pH value increased progressively over time.

The corrosion process of magnesium alloys typically exhibits two distinct modes: uniform corrosion and localized corrosion. These modes differ significantly in their kinetics and underlying mechanisms. During uniform corrosion, the corrosion rate across different regions of the magnesium alloy surface is approximately the same. This process is primarily dominated by the micro-galvanic cell effect, where there is no fixed anode or cathode, and the anodic and cathodic sites alternate dynamically, preventing the formation of a stable polarization zone. Meanwhile, although the passivation film formed in situ can suppress corrosion, its porous structure makes it prone to penetration, leading to continuous localized dissolution. Inoue et al. [64] studied the corrosion behavior of pure magnesium, AZ31, and AZ91E in boric acid buffer solution containing Cl^−^ ions, and the results showed that with the extension of soaking time, the degradation rate of magnesium alloy in NaCl solution containing boric acid buffer was faster than that in solution without boric acid buffer.

Local corrosion is mainly manifested as galvanic corrosion and pitting corrosion [65]. The former is caused by the polyphase of the material, and the standard electrode potential of magnesium is extremely low (−2.37 V), which leads to the anode and cathode formed by magnesium alloy, its internal impurities, and the second phase. The resulting potential difference causes the anode to be corroded and dissolved and produces corrosion by-products around the contact point. And then internal galvanic corrosion occurs. When magnesium alloy and other alloys are in the corrosive liquid and touch, metals or alloy materials such as aluminum, zinc, and iron with higher electrode potential will become the cathode, resulting in strong external galvanic corrosion of magnesium alloy as the anode [66]. Kirkland et al. [67] simulated the research environment at 37 °C, and the results show that the corrosion degree of magnesium alloy at 37 °C is twice as high as that at 20 °C, and the change of surface temperature will accelerate the electrochemical reaction cluster and change the corrosion mechanism. At the same time, the pH value of the human body is about 7.4. When the pH value is lower than 10.5, the corrosion rate of the material will accelerate with the decrease of the pH value, and the sensitivity to local corrosion will also increase. Pitting corrosion usually occurs in some active sites within a small range on the surface of magnesium alloy, which is a local dissolution phenomenon caused by the rupture of metastable passivation film on the surface of magnesium alloy. The development of pitting corrosion can be divided into three stages: nucleation, expansion, and stabilization. The pitting holes formed are small in diameter and difficult to detect, which is a relatively hidden corrosion behavior [68].

Different from vitro experiments, magnesium alloy faces more complex physiological conditions after implantation in organisms, which may be affected by body fluid composition, protein adsorption, local cell reaction, and local pH changes. At the same time, when magnesium alloy is implanted into organisms, it is not only affected by chemical corrosive media but also affected by dynamic mechanical loads in the body. The mechanical stress generated by magnesium alloy may cause surface micro-cracks, thus changing the rate of local corrosion. Therefore, there is a certain difference between in vivo degradation and vitro degradation in simulated body fluids [69,70]. Yang et al. [71] found that there were differences in pH value, ion concentration, and tissue fluid in local tissues, resulting in obvious regional and time-dependent degradation modes in various parts of the body, with the degradation rate ranking in order of intramedullary lumen > intramuscular > cortical bone. This may be due to the continuous erosion of fluid in the bone marrow cavity, which accelerates the dissolution of implant materials, as well as the tissue envelope effect, which slows down the degradation rate of implants in the muscle and bone cortex.

The release of metal ions from corrosion of biodegradable Mg alloys may cause systemic toxicity to humans and local toxicity to the cells surrounding the implant. Normally, within a certain threshold level, the release of toxic element ions in the body can be tolerated by the body, but, at excessive concentrations, it will have adverse effects on the body tissues. The biosafety, biocompatibility and cytotoxicity of magnesium alloy implants have been studied by a large number of relevant researchers, and a large number of animal tests have been performed. Some magnesium base alloys have also passed clinical safety tests [72].

**Figure 2:**
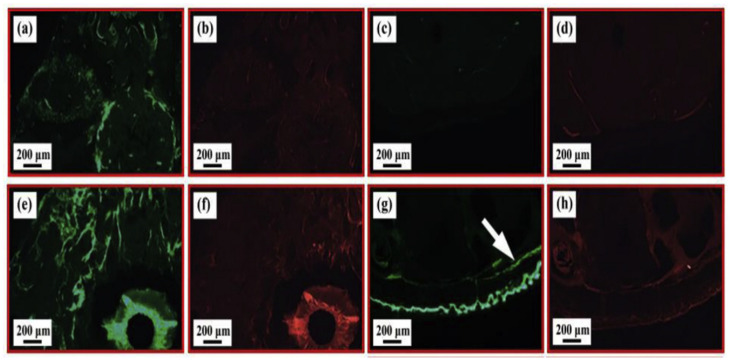
Fluorescence images of a cross section of the distal femur of mice 4 weeks after implantation (**a**–**d**) for the control group without implantation and (**e**–**h**) for the group without Mg-2Sr-Ca alloy. The white arrow indicates the formation of new endosseous bone [75].

Ding [73] manufactured a newly designed Mg-3Zn-0.2Ca alloy clip by using the new route of the process technology combining hot extrusion and blanking and studied the biocompatibility evaluation of this material at different times. The results showed that the clip implanted near the heart degraded faster than the clip implanted far away from the heart. Histological analysis and various blood biochemical parameters at different times after implantation of the clips also confirmed that there was no tissue inflammation around the clips; however, there was a significant fracture behavior on the side near the heart, mainly due to the pulsing stress generated by blood flow that accelerated the degradation of the magnesium alloy surgical clips. Li et al. [74] prepared a binary Mg-Ca alloy and implanted it into the left and right femoral shaft of a rabbit femur. Imaging examination showed that the Mg-1Ca alloy needle gradually degraded in vivo within 90 days, and the newly formed bone could be clearly seen at the 3rd month. There was no significant difference in serum magnesium content at different degradation stages, indicating that the alloy had good biocompatibility. Chen et al. [75] successfully prepared Mg-2Sr-Ca alloy and conducted in vivo degradation tests on it, as shown in Figure 2, which shows the corresponding fluorescence images of the cross section of the distal femur of mice, with new bone formation in the diaphysis and trabecular regions. Compared with the control group, Mg-2Sr-Ca alloy had more new bone trabecula formation.

Wang et al. [76] implanted hydroxyapatite (Ca-def HA)-coated Mg-Zn-Ca alloy into rabbit femur and observed its dynamic degradation behavior in vivo by Micro-CT system during the implantation period of 24 weeks, indicating that the effective life of the coating in vivo is about 8 weeks. After that, the degradation rate of the coated implant increased significantly. Histopathological examination showed that Ca-def HA coating had good bone conductivity and was conducive to the formation of more new bone on the magnesium alloy surface. Zhang et al. [77] studied a binary Mg-Zn magnesium alloy as a biodegradable biomedical material. When this Mg-Zn alloy material was implanted into the femoral shaft of rabbits, radiographs showed that magnesium alloy could be gradually absorbed in the body at a degradation rate of about 2.32 mm/year, and the hematoxylin- and eosin (HE)-stained sections around the Mg-Zn rod indicated newly formed bone around the implant. The stained tissues included heart, liver, kidney, and spleen tissues, and biochemical measurements including serum magnesium, serum creatinine (CREA), blood urea nitrogen (BUN), glutamate-pyruvate aminotransferase (GPT), and creatine kinase (CK) demonstrated that the degradation of Mg-Zn in vivo did not damage vital organs. In addition, no adverse effects of hydrogen produced by degradation were observed, and no negative effects due to zinc release were detected. These results indicate that the novel Mg-Zn binary alloy has good biocompatibility in vivo.

## 3. Coatings Classification

### 3.1. Inorganic Coatings

#### 3.1.1. Micro-Arc Oxidation Coatings

**Figure 3:**
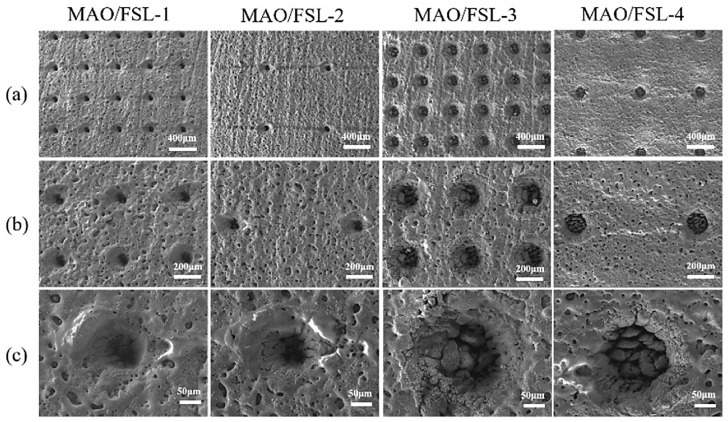
Morphological characteristics of MAO/FSL coating. (**a**–**c**) SEM images with different magnification [32].

Micro-arc oxidation coating is an electrochemical treatment that forms a ceramic film on the surface of metals such as aluminum, magnesium, and titanium. By applying a high-voltage electric field to the surface of the metal, microarc discharge occurs on the surface, thus forming a dense ceramic oxide film on the surface of the metal [78]. The coating has the characteristics of high productivity, ecological friendliness, economic efficiency, good wear resistance and corrosion resistance, high hardness, excellent bonding strength with the substrate, etc. Due to these advantages, MAOC has become an international research hotspot and is widely used to improve the surface properties of magnesium alloys [79]. In recent years, research on MAOC continues. Wang et al. [32] prepared a coating with micro and nano structures on the surface of magnesium alloy AZ31B by micro-arc oxidation (MAO) technology and further treated it by femtosecond laser to regulate its degradation rate. The thickness of the MAO coating is approximately 21 microns. Meanwhile, the critical load determined by the scratch test is 9.5 N, which indicates that the bonding force between the coating and the substrate is approximately 9.5 N. It contains Mg, Ag_2_O, CaCO_3_, and TiO_2_ phases. By femtosecond laser treatment, the coating forms holes with different apertures and spacing (Figure 3), which can effectively control the degradation rate of magnesium alloy and improve its corrosion resistance. Experiments have shown that the MAO/FSL-2 sample (pore size 50 microns, pore distance 1 mm) has the most reasonable degradation rate and that the coating consistently releases Ag^+^, has an antibacterial rate of 99.82%, is non-toxic to osteoblasts, has good biocompatibility and antimicrobial properties, and is promising for use as a new generation of biodegradable implant materials.

**Figure 4:**
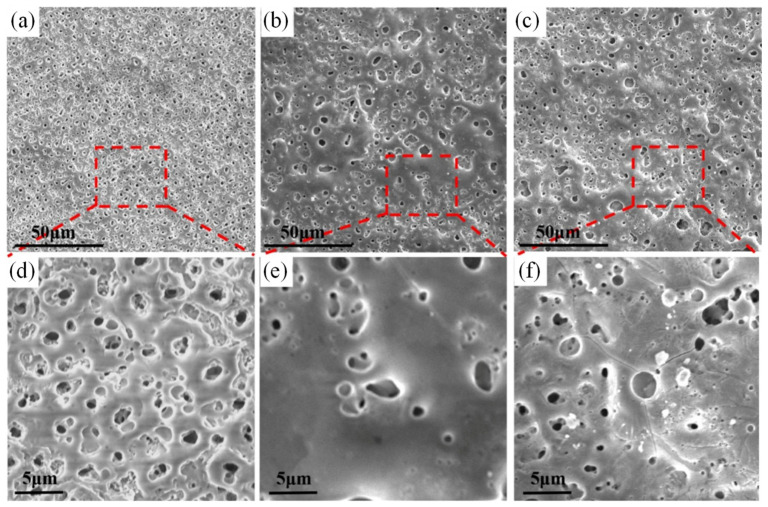
Surface morphologies of wool coatings with different concentrations of HA: (**a**,**d**) 0 g/L, (**b**,**e**) 1 g/L, (**c**,**f**) 2 g/L [80].

Golshirazi et al. [33] significantly enhanced the corrosion resistance of AZ91 magnesium alloy by employing a dual-layer coating system consisting of MAO and a polyethylenimine/κ-carrageenan (PEI/KC) self-assembled layer. The MAO process, conducted in a silicate–fluoride electrolyte, generated a ceramic coating composed of MgO, Mg_2_SiO_4_, and MgF_2_. The subsequent PEI/KC layer effectively filled the pores of the MAO coating, improving mechanical interlocking and adhesion strength while further preventing the penetration of corrosive media. This bilayer coating system demonstrates promising potential for biomedical applications. In order to study the degradation behavior and biomedical properties of magnesium alloy, Dong et al. [80] prepared a hydroxyapatite (HA)-enhanced composite coating on the surface of Zn-Ag-Mg alloy by micro-arc oxidation (MAO) technology and investigated its degradation behavior and biomedical properties. The research shows that by adding low-concentration HA particles (1 g/L) to the electrolyte, the thickness of the coating—which is mainly composed of ZnO and HA—is about 10 μm. The corrosion resistance of the alloy is significantly improved, and the corrosion current density is reduced by one order of magnitude (from 4.51 × 10^−5^ A/cm^2^ to 1.06 × 10^−6^ A/cm^2^). The corrosion rate is reduced by more than ten times (0.532→0.012 mm/a). However, the high concentration of HA (2 g/L) will lead to the increase of cracks in the coating (Figure 4) and weaken the structural stability. The 30-day immersion experiment showed that the MAO-HA coating promoted Ca-P precipitation and enhanced bone integration, and the coating containing HA had better surface protein adsorption. The study demonstrates the potential of a low-concentration HA-MAO coating in balancing corrosion resistance, structural stability, and biological activity and provided an optimal strategy for degradable Mg-based biomedical materials.

Qian et al. [81] developed a biodegradable PTMC-MAO composite coating. By combining the ceramic layer generated by micro-arc oxidation (MAO) with a polytrimethyl carbonate (PTMC) polymer layer, the coating significantly inhibits the rapid degradation of magnesium alloy. The experimental results show that the corrosion current density of the composite coating decreases by 851 times to 5.3 nA/cm^2^, and the protection efficiency reaches 99.9%. After 21 days of immersion, the pH change is only 0.25, and the release of magnesium ions is 42 μg/mL, which is much better than that of bare alloy. The coating surface is uniformly degraded without local pitting corrosion, and the MAO microporous structure can be used as a drug carrier to achieve slow release. This technology provides a new strategy for long-term functional maintenance of degradable magnesium alloy implants with excellent corrosion protection and biocompatibility. These studies collectively demonstrate the versatility and effectiveness of MAO-based surface treatments in improving the performance of magnesium alloys for biomedical use. 

Diamond-like carbon (DLC), due to its high hardness, good adhesion, low coefficient of friction, and chemical inertness, can enhance the wear resistance and corrosion resistance of magnesium alloy surfaces. Cui et al. [34] developed a PDMS-modified dual MAO/DLC coating process prepared on the surface of AZ31B magnesium alloy. The composite coating was constructed by MAO and DLC deposition and then hydrophobically modified with polydimethylsiloxane (PDMS). Experiments show that after PDMS penetrates into the pores of the coating, it can improve the density, adhesion, wear resistance and corrosion resistance of the coating, significantly reduce the friction coefficient and corrosion current density, and demonstrate excellent anti-corrosion and lubrication effects, making it suitable for long-term protection of magnesium alloys. Ning et al. [82] prepared different element-doped DLC composite coatings (including H-DLC, Si-DLC, and Cr-DLC) on the surface of AZ31B magnesium alloy by MAO and unbalanced magnetron sputtering. It can be seen from the experimental results that all DLC coatings have improved the tribological properties. Among them, Si-DLC shows the lowest coefficient of friction and wear rate, and H-DLC has the best electrochemical corrosion resistance, but its long-term corrosion resistance is not as good as that of the MAO coating. The research provides multiple feasible paths for the surface protection of magnesium alloys. These studies collectively demonstrate the versatility and effectiveness of MAO-based surface treatments in improving the performance of magnesium alloys for biomedical use. Whether by incorporating antibacterial agents like silver, enhancing biological activity with hydroxyapatite, or applying biodegradable polymer composites, MAO coatings show strong potential in achieving a balance between degradation control, biocompatibility, and mechanical stability. This lays a solid foundation for the development of next-generation implant materials.

#### 3.1.2. Phosphate Coatings

Nowadays, chromate coatings are found to show excellent corrosion resistance and self-healing ability, but because the coating will release Cr^6+^, which is toxic to the human body, the use of this coating is highly limited. As the corrosion resistance of phosphate-based conversion coatings is roughly the same as that of chromate-based coatings, and as a good, cost-effective, and environmentally friendly coating, it has become a current research focus [83]. For example, Zhen et al. [84] made two kinds of phosphate chemical conversion coatings by different methods: phosphate conversion coatings (Im-PCCs) for conventional impregnation treatment and phosphate conversion coatings (Br-PCCs) for brush conversion treatment. Im-PCCs is formed by immersion treatment. The coating can effectively inhibit the evolutionary reaction of hydrogen, reduce the corrosion current density, and improve the corrosion resistance. However, with the extension of the treatment time, the electrical contact resistance will also increase. Compared with the previous coating, Br-PCCs, which are formed by brush coating, have a more uniform microstructure, significantly reducing the electrical contact resistance, improving the electrical conductivity, and effectively inhibiting corrosion. By optimizing the ratio of strong oxidizer KMnO_4_ (3.0 g /L), additive Na_2_MoO_4_ (1.5 g/L)n and surfactant OP-10 (1.0 g/L) in the phosphatic solution, Zhang et al. [35] prepared a phosphate conversion coating with both electrical conductivity and corrosion resistance on the surface of AZ91D magnesium alloy. The coating has a double-layer structure, where β phase protrusions form conductive points and KMnO_4_ inhibits crystal formation and promotes the formation of extremely thin oxide film, making the contact resistance as low as 4.91 Ω, reducing the corrosion current density by more than one order of magnitude, and significantly improving the comprehensive properties of magnesium alloy. Jayaraj et al. [85] developed a two-stage chemical conversion coating technology based on cerium phosphate to improve the corrosion resistance and biocompatibility of AZ31 magnesium alloys. Cerium oxide/hydroxide composite coating (CePW) and cerium phosphate rich coating (CePE) were obtained by pre-deposition of a magnesium phosphate layer and secondary treatment in water or ethanol-based cerium nitrate solution, respectively. The results showed that CePE showed better corrosion resistance (corrosion rate 0.92 mm/y) in both NaCl and simulated body fluids, while CePW had self-healing properties due to the cerium oxide/hydroxide content. Both coatings showed good cellular compatibility (cell survival > 75%) and are suitable for engineering and biomedical applications, especially for biodegradable implants.

Water is an important factor causing electrochemical corrosion on the surface of magnesium alloys, so hydrophobic treatment is an effective measure to improve the surface corrosion resistance of magnesium alloys. Xia et al. [86] prepared a superhydrophobic phosphate chemical conversion coating (PCC-SHS) on the surface of magnesium alloy by combining biomimetic superhydrophobic technology and phosphate chemical conversion technology. The surface defects of the modified coating are effectively blocked, the film layer is denser, the static water contact angle is as high as 156.5°, and the rolling angle is as low as 5°, showing excellent superhydrophobic properties. Electrochemical tests show that the corrosion current density of the PSC-SHS coating is reduced by one order of magnitude, the low-frequency impedance modulus is increased by one order of magnitude, and the high impedance value can still be maintained after 12 days of immersion, showing efficient and durable corrosion-protection performance. The neutral salt spray test shows that the salt spray resistance of the PSC-SHS coating exceeds 100 h. The coating has excellent super hydrophobicity and self-cleaning properties, providing efficient protection for magnesium alloys.

#### 3.1.3. Fluorinated Coatings

In addition to phosphate-based conversion coatings, fluorine-containing biomedical coatings on magnesium alloys have been studied by other authors [87]. Barajas et al. [88] studied the microstructure and biodegradation mechanism of fluoride conversion coating of AZ31 magnesium alloy formed in hydrofluoric acid (HF) solution. It was found that the coating, which is mainly composed of magnesium hydroxyl fluoride, can significantly improve the corrosion resistance of AZ31 alloy in Hanks’ solution. The thickness and fluorine ion content of the coating increase with the increase of HF concentration and treatment time, but cracks and defects also appear in the coating. These defects are mainly caused by the dissolution of the AlxMny intermetallic phase in the alloy in HF solution. The corrosion resistance of the coating increases with the increase of fluorine ion content, but after a long time soaking, the protective performance of the coating will decline, resulting in corrosion of the base alloy. The results show that the microstructure of the alloy has an important effect on the formation and corrosion mechanism of the coating. Subsequently, Dzikova et al. [36] successfully prepared an MgF_2_ coating on an AZ31 magnesium alloy surface by soaking Na[BF_4_] molten salt for a different time and temperature (430 °C and 450 °C). The study shows that the coating thickness and corrosion performance increase with the increase of treatment temperature and time, but there are pore defects on the coating surface, and it is also believed that these defects may be related to the AlxMny intermetallic reaction in the matrix. It was also found that the microstructure of the coating, such as grain size and defect distribution, significantly affect its corrosion resistance. The immersion test showed that the coating significantly reduces the corrosion rate of AZ31 magnesium alloy, but the defects still lead to local corrosion.

#### 3.1.4. Hydrothermal Coatings

**Figure 5:**
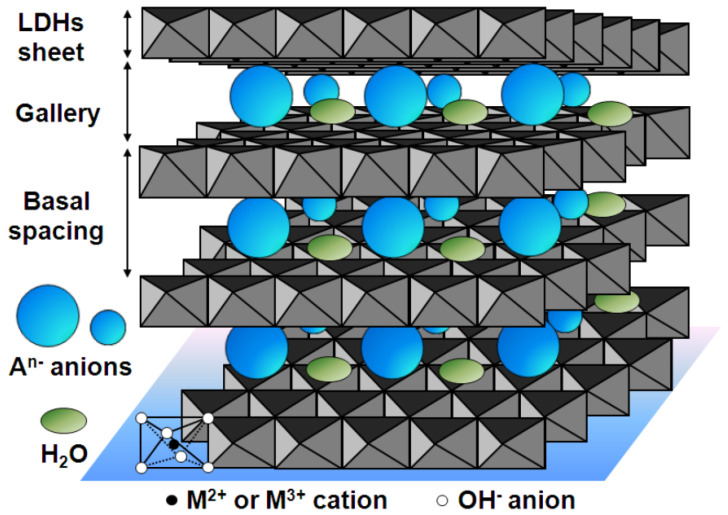
Schematic diagram of layered double hydroxide (LDH) structure [91].

Hydrothermal coating is a coating formed by hydrothermal method under high temperature and high pressure, using water as the solvent to cause the reactants to react on the surface of the substrate. The preparation process includes the construction of the reaction system, the use of a high-pressure reactor for the heating reaction, and the reaction post-treatment steps [89]. Layered double hydroxides (LDHs), also known as talc-like compounds, are also anion exchangers [90]. Their typical structure is shown in Figure 5 [91]. As a typical material manufactured by hydrothermal coating, LDH has the characteristics of good uniformity, high crystallinity, strong controllability, and environmental friendliness and has been widely used in energy storage, catalysis, biomedicine, and optical devices.

**Figure 6:**
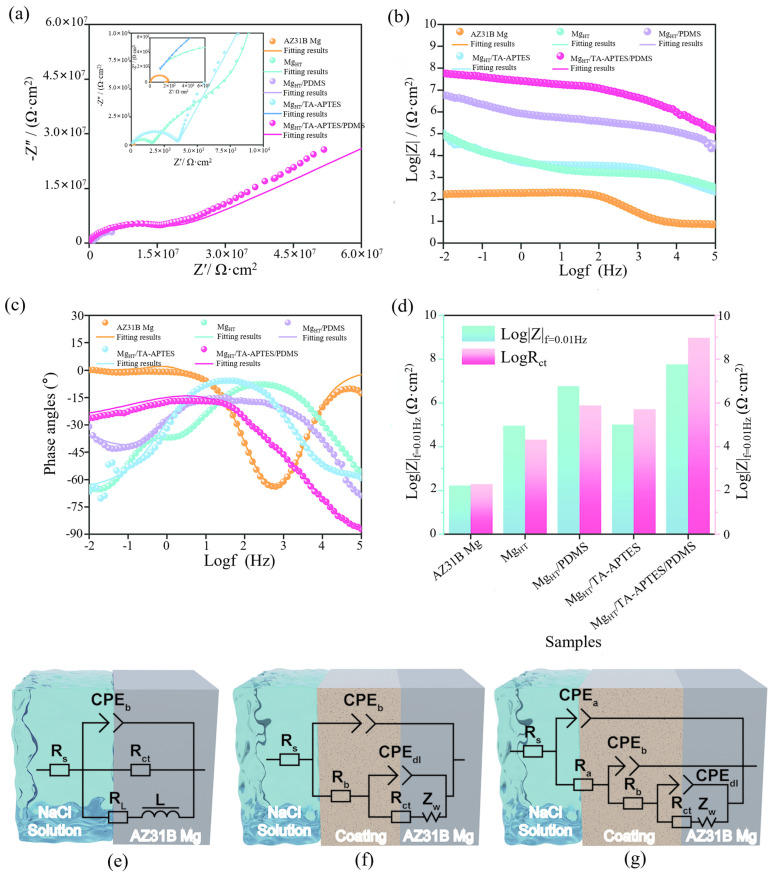
EIS spectra of naked AZ31B magnesium, MgHT, MgHT/PDMS, MgHT/TA-APTES, and MgHT/TA-APTES/PDMS. Nyquist diagram and magnified Nyquist diagram (**a**). |Z| Bode plot (**b**) for frequencies. Bode plot (**c**) of phase angle against frequency. Histogram (**d**) of log Rct and log|Z|f = 0.01Hz values for different samples. Used to fit the equivalent circuit models of bare AZ31B magnesium alloy (**e**), MgHT/PDMS and MgHT/TA-APTES (**f**), and MgHT/TA-APTES/PDMS (**g**) [37].

At present, a few people have adopted the method of combining hydrothermal treatment with other coatings to improve the performance of magnesium alloy substrates. For example, Cheng et al. [37] combined a tannic acid (TA)-amino-silane (APTES) complex with a polydimethylsiloxane (PDMS) coating by hydrothermal treatment. To develop a new superhydrophobic magnesium alloy (Mg_HT_ /TA-APTES/PDMS), a rough layer of magnesium hydroxide is formed on the surface of magnesium alloy AZ31B by the hydrothermal method, and the micro–nano structure is constructed by the reaction of TA and APTES with a Michael addition and Schiff base. PDMS reduces the surface energy, and the superhydrophobic properties are finally achieved (contact Angle 165.4°, slide Angle 3.4°). The corrosion current density of the coating is as low as 9.931 × 10^−11^ A/cm^2^, the corrosion rate is significantly reduced, and the coating shows excellent chemical stability (resistance to acid, alkali, and salt corrosion) and mechanical durability (resistance to water impact and sandpaper wear). Electrochemical impedance spectroscopy and the polarization curve (Figure 6) confirm that the synergic anticorrosion mechanism is due to the isolation of the air layer and the density of the coating.

Aiming at the corrosion resistance and antibacterial properties of magnesium alloy, Zerankeshi et al. [92] constructed a magnesium hydroxide protective coating on the surface of Mg-2Ag alloy by the hydrothermal method. By comparing cast state (AC) alloy with solid solution state (ST) alloy, it is found that the coating of AC alloy is porous and easy to crack due to the dendrite structure and Ag-enriched phase, and the corrosion current density is as high as 84.52 μA/cm^2^. And after 400 °C heat treatment, microstructure homogenization, silver phase dissolution, and the formation of dense uniform Mg(OH)_2_ coating (thickness 142.6 μm), ST alloy corrosion current density decreased to 1.84 μA/cm^2^, corrosion rate decreased from 1.93 mm/year to 0.04 mm/year. Electrochemical polarization and a hydrogen release test show that the coating significantly prolongates the corrosion latency (ST up to 140 h) and causes no obvious damage to the surface after corrosion. However, Gerashi et al. [93] studied the effect of a hydrothermal coating on the degradation behavior and biocompatibility of Mg-4Zn-0.3Sr alloy. The Mg(OH)_2_ coating was prepared on the surface of the alloy by the hydrothermal method at 160 °C, and it was found that the homogeneous 120 μm thick coating without cracks could be formed after treatment for 5 h. The coating reduced the corrosion current density from 2.58 μA/cm^2^ to 0.48 μA/cm^2^ (81% reduction) and the hydrogen release rate by 90%. Cell experiments showed that the coating significantly improved the adhesion and survival rate of L929 fibroblasts, which was attributed to the reduced degradation rate and enhanced surface stability, confirming its potential as a medical implant material.

#### 3.1.5. Bioceramic Coatings

Bioceramic coating is a method of applying biologically active or inert ceramic materials onto the surface of biometals, aiming to enhance their biocompatibility, corrosion resistance, and bone-bonding ability. Currently, the most widely used ceramic coating in metal implants is hydroxyapatite (HA), which is rich in phosphorus and calcium, the main elements for bone synthesis in the human body. It can promote the formation of new bone in the human body and is thus widely applied in artificial bones, teeth, joints, and other artificial implant products [94]. Hernández et al. [38] investigated the preparation of biomimetic HA coatings on pure magnesium surfaces and their physiological corrosion behavior. HA coatings were successfully deposited on the surface of pure magnesium by using supersaturated calcification solution (SCS) through an improved biomimetic deposition method. Research shows that the corrosion behavior of the coating is affected by surface pretreatment and deposition time. The 6 h deposited coating after heat treatment exhibits higher corrosion resistance and is expected to be used in the development of new degradable orthopedic implants. Jiang et al. [39] synthesized HA coatings on the surface of magnesium alloys by using the ammonium polyphosphate (PPA)-assisted hydrothermal method. The introduction of PPA significantly improves the compactness and uniformity of the coating, enhances mechanical properties, and improves corrosion resistance. Compared with traditional HA coatings, PPA-HA coatings have higher adhesion, better hardness and elastic moduli, and demonstrate excellent long-term corrosion resistance in in vitro experiments, showing broad application prospects for the protection of degradable magnesium-based implant materials. Rahman et al. [40] constructed a dense and crack-free coating structure on WE43 magnesium alloy by combining two layers of Ca-P coatings (DCPD and HA) through anodic oxidation. The HA coating is converted from DCPD through alkaline treatment and exhibits excellent microstructure, adhesion strength, and corrosion resistance. Experiments show that the HA-WE43 sample has the lowest hydrogen release rate, corrosion rate, and mass loss, and its corrosion life in simulated body fluids can reach 384.7 days, demonstrating its application potential in biomedical implant materials. Zaludina et al. [41] successfully prepared calcium phosphate (CaP) coatings on pure magnesium substrates by using a simple chemical conversion method, which was achieved through impregnation. The first coating was DCPD, and the second coating was treated with an alkaline solution to obtain HA. Both coatings significantly enhanced the corrosion resistance of magnesium substrates. Among them, the DCPD coating demonstrated superior corrosion resistance due to its low porosity, thickness, and good coverage. Liao et al. [95] constructed microemulsion cone structures on the surface of magnesium alloys by femtosecond laser technology, significantly enhancing the density, thickness, and bonding strength with the substrate of HA coatings. Experiments show that the corrosion rate of the HA coating treated by femtosecond laser in simulated body fluids is only 0.146 mm/year, and it can rapidly induce bone tissue mineralization and promote osteoblast proliferation, providing strong support for the application of magnesium alloys in clinical bone implant materials.

Plasma spraying technology is an advanced thermal spraying process. It heats powder materials to a molten or semi-molten state through a high-temperature and high-speed plasma flame flow and then sprays them onto the surface of the substrate to form a coating. It can handle high-melting-point materials, form high-quality coatings, and has good bonding strength and low porosity. Bugdayci et al. [42] prepared HA coatings on the surfaces of AZ31 and AZ91 magnesium alloys by plasma spraying technology to enhance their corrosion resistance as degradable implant materials. The experimental results show that the HA coating significantly reduces the corrosion rate of the alloy in the simulated fluid, from 1.2 mm/year without coating to 0.4 mm/year. SEM and XRD analyses confirm that the coating was uniform and porous, with a thickness of up to 120 microns, and free of impurities. Corrosion tests show that the coating reduces the weight loss of AZ91 from 123.1 mg to 38.8 mg within 504 h, enhancing the protective effect by 3.1 times. Bansal et al. [43] prepared HA coatings doped with different proportions (4%, 8%, 12%) of Sr on the surface of AZ31 magnesium alloy by plasma spraying technology. Research findings indicate that as the Sr content increases, the surface hardness of the coating significantly rises, the surface roughness decreases, and the coating exhibits superior hydrophilicity and corrosion resistance. Electrochemical tests show that the corrosion current density of the HA + 12%Sr coating is the lowest, demonstrating the best corrosion resistance. In addition, the immersion test of the coating in simulated body fluids further confirms its excellent anti-corrosion stability and biological activity. These results indicate that the HA/Sr coating can significantly improve the surface properties and corrosion resistance of AZ31 magnesium alloy and is expected to become a promising coating material for future bone implant applications.

### 3.2. Organic Coatings

Organic coatings are widely used in the surface treatment of biodegradable magnesium alloys to enhance their corrosion resistance and biocompatibility. According to the sources and properties of the coating, they can be divided into two major categories: synthetic polymer coatings and natural polymer coatings. Synthetic polymer coatings usually have good mechanical strength and controllable degradability, while natural polymer coatings show great application potential in the biomedical field due to their excellent biocompatibility and biodegradability.

#### 3.2.1. Synthetic Polymer Coatings

**Figure 7:**
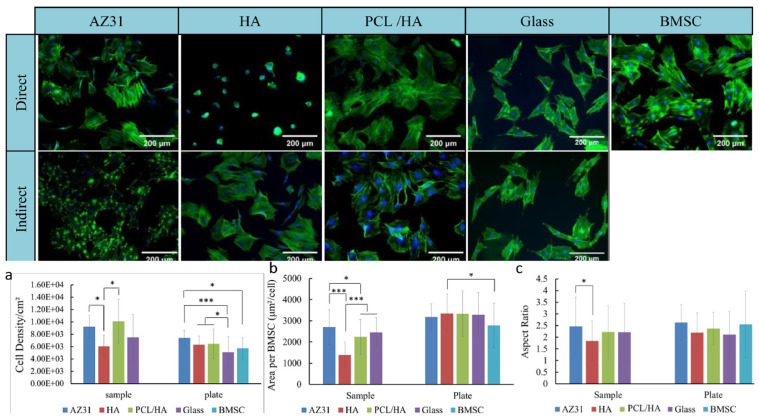
Fluorescence images of BMSC after 24 h of in vitro culture, as well as (**a**) adhesion density of BMSC, (**b**) diffusion area of each BMSC, and (**c**) aspect ratio of BMSC on the sample surface (in direct contact with the sample) and on the culture plate around each corresponding sample (in indirect contact with the sample). * *p* < 0.05, *** *p* < 0.001 [44].

Biodegradable polymer coatings are films or coverings composed of natural or synthetic polymer materials that are attached to the surface of the substrate through physical or chemical methods. They can undergo controllable degradation under preset conditions and eventually transform into environmentally friendly products. At present, the most commonly used degradable polymers are polylactic acid (PLA), polycaprolactone (PCL), polyglycolic acid (PGA), PLGA copolymers, etc. [96]. With the advancements in synthetic biology, nanotechnology, and green chemistry, such coatings will play a more crucial role in precision medicine, circular economy, and ecological protection. In recent years, some scholars have also conducted research on this aspect. For instance, Zhang et al. [44] significantly enhanced the corrosion resistance and biocompatibility of the material by preparing a polycaprolactone/hydroxyapatite (PCL/HA) composite coating on the surface of AZ31 magnesium alloy. PCL fills the pores of HA crystals to form a dense structure, which enhances the bonding strength of the coating and reduces the electrochemical corrosion rate to 6.9 mm/year (23 times lower than that of uncoated magnesium alloys). After 38 days of external immersion, the coating remained stable. Cell experiments have shown that the PCL/HA coating is more conducive to the adhesion and proliferation of bone marrow mesenchymal stem cells (BMSC) (Figure 7), but its antibacterial performance is insufficient, and it has no significant inhibitory effect on methicillin-resistant Staphylococcus aureus (MRSA). This coating has application potential in biodegradable implant materials, but its antibacterial performance needs to be further optimized.

Grewal et al. [45] studied a new type of self-healing coating (PA-PCL caps) and found that this coating could be used for corrosion inhibition of degradable magnesium alloys. By encapsulating phytic acid (PA) in PCL microcapsules, a hybrid coating with a honeycomb core structure is formed. Experiments show that this coating exhibits significant hydrophobicity (contact Angle 116.3°) in simulated body fluid (SBF), with a corrosion potential of −0.28 V, a corrosion current density as low as 1.1 × 10^−9^ A/cm^2^, and a corrosion rate of only 2.5 × 10^−4^ mm/year. When the coating is damaged, PA releases and promotes the mineralization of hydroxyapatite (HA) by chelating Mg^2+^ and Ca^2+^, repairs the scratch within one day, and restores the hydrogen release rate (0.36 mL/cm^2^/day) and pH value (7.10). In addition, the increase in pH value and the increase in Mg2 concentration respectively increased the PA release by 2.5 times and 3.1 times, further enhancing the self-repairing ability of the coating.

Parylene C is a polymer with excellent biocompatibility, low water permeability, and durability and is often used as a coating for biomedical implant devices. Surmeneva et al. [46] prepared 2-micron-thick Parylene C coatings on the surfaces of AZ31, WE43 and AZ91 magnesium alloys by chemical vapor deposition. The results show that the organic coating significantly enhances the corrosion resistance of the alloy. In the simulated fluid, the corrosion current density decreases and the polarization resistance increases. Mechanical tests show that the coating has an appropriate elastic modulus (4.19–5.14 GPa) and a good elastic recovery rate (40–48%). Surface analysis confirms that the coating is uniform and dense, with a crystalline size of 4.9 nm. Research confirms that Parylene C can effectively delay the degradation of magnesium alloys while maintaining mechanical adaptability, demonstrating application potential in the field of degradable implantable devices.

#### 3.2.2. Natural Polymer Coatings

Natural polymer coatings such as chitosan, hyaluronic acid, collagen, and cellulose coatings have the advantages of good biocompatibility, environmental friendliness, degradability, and antibacterial properties. At the same time, they do not produce acidic products like synthetic polymers do, thus not causing a series of inflammatory responses in the body [97].

Chitosan is a natural polysaccharide with good biocompatibility and antibacterial properties. It forms chemical bonds or physical adsorption with the surface of magnesium alloys through amino groups, which can effectively improve the corrosion resistance and antibacterial performance of magnesium alloys. Moreover, it also contains glycosaminoglycans, the main component of the extracellular matrix [98]. Based on this, chitosan has been widely used in the medical field. de Sousa Santos et al. [47] developed a sustainable smart coating based on chitosan and layered dihydroxide (LDH) to enhance the corrosion resistance of AZ31 magnesium alloy. In the study, natural inhibitors (vanalic acid, gallic acid, and citrate ions) were loaded into LDH and then dispersed into the chitosan matrix. Experiments show that this intelligent coating can trigger the release of corrosion inhibitors at different pH values, and the coating containing gallic acid ions significantly improves the corrosion resistance of magnesium alloys; this is expected to be applied in the biomedical and automotive fields. Manzur et al. [99] reported an intelligent coating based on chitosan and layered dihydroxide (LDH) for enhancing the corrosion resistance of AZ31 magnesium alloy. In the study, LDH was synthesized by co-precipitation and loaded with natural inhibitors (vanalic acid, gallic acid, and citrate ions). The author found through experiments that this intelligent coating can respond to pH changes to release inhibitors, significantly improving the corrosion resistance of magnesium alloys. Especially, the coating containing gallic acid ions shows excellent anti-corrosion effects.

Hyaluronic acid (HA) is a polymer derived from an organism, so the side effects on the human body are small. At the same time, it has good antibacterial properties and is one of the major components of extracellular matrix, at the same time involved in cell adhesion, proliferation, and differentiation [100]. Agarwal et al. [48] improved the corrosion resistance and biocompatibility of bioabsorbable magnesium through plasma electrolytic oxidation (PEO) treatment and coating with hyaluronic acid (HA) and its derivatives. PEO forms a porous oxide film on the surface of magnesium, but it is brittle and prone to local corrosion. In the study, the magnesium surface after PEO was treated with HA and carboxymethyl cellulose (CMC), which increased the hydrophilicity of the surface, increased the surface roughness, reduced the contact angle, and improved the cell activity and the ability of new bone formation. It was found through experiments that the HA coating significantly improved the initial corrosion resistance and had a self-healing ability that could quickly self-repair the damage in the scratch test. Kim et al. [101] investigated the functions of hyaluronic acid and its derivative coatings and the hydroxide film on bioabsorbed magnesium. Research has found that the HA coating can significantly enhance the initial corrosion resistance of magnesium and achieve rapid self-repair through cross-linking with carboxymethyl cellulose. Experiments show that the HA coating can effectively inhibit corrosion in vitro and promote the proliferation and differentiation of osteoblasts. In in vivo experiments, magnesium screws coated with HA demonstrate excellent bone defect repair capabilities and significantly delay the degradation process of the screws.

### 3.3. Composite Coatings

#### 3.3.1. Sol-Gel Coatings

Sol-gel coating is prepared by the sol-gel method, and the preparation method is usually either organic or inorganic [102]. Organic methods generally involve the dissolution of monomer or metal-like alkaloid precursors in alcohol or organic solvents [103]. Inorganic methods, on the other hand, form a network by gelating suspended colloidal particles in the size of 1–1000 nm in a continuous liquid phase [104]. The sol-gel process is a wet chemical process that involves three basic stages, namely hydrolysis, polycondensation, and thermal densification [105]. The formation of sol-gel involves four stages: (a) hydrolysis stage, (b) condensation and polymerization of monomers to form chains and particles, (c) particle growth, and (d) agglomeration of polymer structures, followed by the formation of a network in the liquid medium to increase viscosity, followed by drying to obtain the gel [106]. Among the different current technologies, the sol-gel method has received a lot of attention due to its versatility, simplicity, time, and cost effectiveness [107].

Currently, silane sol-gel coatings are widely used as antiseptic and biocompatible coatings on Mg alloys in orthopedic applications due to their hydrophobic Si-O-Si network, low sensitivity to galvanic reactions with Mg, ease of chemical modification, high adhesion properties, and low cytotoxicity [108]. For example, Merino et al. [49] investigated the enhancement of corrosion resistance of AZ31B magnesium alloy by anodic oxidation and epoxy alkylsilane sol-gel coating. Firstly, through anodizing treatments at different voltages, it was found that the oxide film obtained after 2 min of treatment at 100V was rich in MgO and had good corrosion resistance. Subsequently, the protection effect was further improved by the epoxy alkylsilane sol-gel coating with the addition of SiO_2_ nanoparticles. The results show that the coating can significantly improve the corrosion resistance of magnesium alloy at both 110 °C and 160 °C curing temperatures, and the protection efficiency of 110 °C-cured coatings reaches 98.6% after 72 h immersion. Qian et al. [50] prepared a hybrid coating with 3-epoxy-propoxy-trimethoxy-silane (GPTMS) and tetraethoxy-silane (TEOS) as precursors by the sol-gel method to improve the corrosion resistance of magnesium alloys. The results show that the coating has good barrier properties and can significantly improve the corrosion resistance of magnesium alloy. The morphology and chemical structure of the coating were analyzed by SEM and FTIR. The results show that the coating is uniform, compact, and well-combined with the substrate. The polarization curve and EIS show that the coating can reduce the corrosion current density by three orders of magnitude, showing excellent anti-corrosion properties. However, with the extension of soaking time, the covalent bonds in the coating gradually hydrolyze, resulting in decreased barrier properties and weakened corrosion resistance.

**Figure 8:**
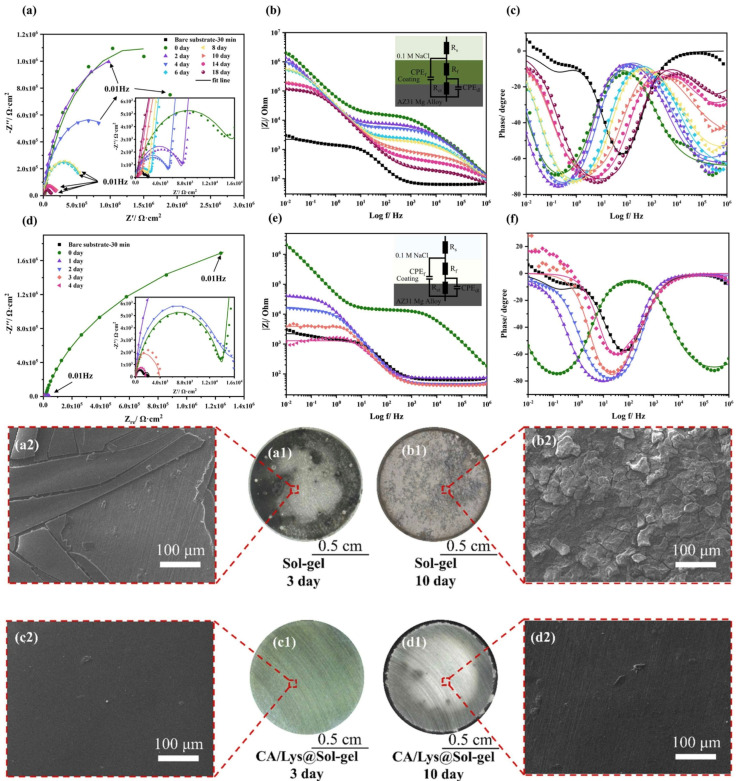
Nyquist and Bode diagrams of CA/Lys@sol-gel (**a**–**c**) and sol-gel (**d**–**f**) (Black lines with black rectangular squares indicate bare AZ31 soaked in 0.1 M NaCl after 30 min) and surface morphology during corrosion process: macroscopic or microscopic morphology of sol-gel soaked in 0.1 M NaCl for 3 days (**a1**,**a2**); The macro- or micro-morphology of sol-gel soaked in 0.1 M NaCl for 10 days (**b1**,**b2**); Macroscopic or microscopic morphology of CA/Lys@sol-gel immersed in 0.1M NaCl for 3 days (**c1**,**c2**); Macroscopic or microscopic morphology of CA/Lys@sol-gel immersed in 0.1 M NaCl for 10 days (**d1**,**d2**) [52].

A sol-gel film alone does not provide long-term protection for magnesium from corrosion in a saline solution [109]. Therefore, in order to obtain a more durable anti-corrosion coating, Li et al. [51] studied a sol-gel coating modified by levodopa (DOPA) to enhance the anti-corrosion properties of magnesium alloy AZ31. The micro–nano defects in the sol-gel were filled by DOPA self-polymerization, which significantly improved the densification and corrosion resistance of the coating. The experimental results showed that the DOPA-modified coating could provide long-term corrosion protection for more than 14 days in 0.1M NaCl solution, while the unmodified coating could only maintain 2–3 days. The optimal DOPA concentration of 2 mg/mL was determined, and the structure and properties of the coating were verified by a variety of characterization techniques. Meanwhile, Li et al. [52] proposed a novel sol-gel coating based on the polymerization modification of catechol (CA) and lysine (Lys) to enhance the corrosion resistance of magnesium alloy AZ31. The study showed that the coating filled the micro–nano pores of the sol-gel through the co-deposition of CA/Lys, formed a dense structure, and could effectively block the penetration of Cl^−^ and water. In 0.1 M NaCl solution, the thickness of the modified coating is only 9 μm, but it can provide protection for up to 18 days, while the unmodified coating only lasts for 3 days. Optimizing the process (CA/Lys concentration of 4 mg/mL, treatment time of 11 h) caused the coating to show excellent electrochemical impedance (10^5^ Ω·cm^2^) and slow release hydrogen performance. As can be seen from Figure 8, the corrosion resistance of the modified coating was significantly better than that of the unmodified coating. Characterization confirms that CA/Lys is environmentally friendly by bonding to silane networks via hydrogen and chemical bonding (CA toxicity is lower than chromate). This study provides a new strategy for long-term anticorrosion of lightweight magnesium alloys.

Durán et al. [53] developed a multilayer hybrid sol-gel coating based on the TEOS-GPTMS system and applied it to the surface of Elektron 21 magnesium alloy, which has the dual functions of a corrosion-resistant barrier and biological activity. The inner barrier coating inhibits the corrosion of magnesium alloy in simulated body fluids (SBF) by silane precursors (TEOS and GPTMS), and the outer layer is doped with calcium (Ca) and magnesium (Mg) to promote biological activity. The experimental results show that the multilayer coating can reduce the corrosion current density of the alloy by three orders of magnitude and improve the corrosion resistance significantly. The introduction of Ca and Mg promote the growth of the apatite phase, but excessive salt would cause the coating to become porous, accelerating electrolyte penetration and substrate deterioration. The study confirmed that the TEOS–GPTMS hybrid system is an effective method to construct dual-function coatings, but the salt content and aging time need to be optimized to balance corrosion protection and biological activity.

Talha et al. [110] prepared a mixed silane coating based on γ-epoxy-propoxy-trimethoxy-silane (GPTMS), tetraethoxy-silane (TEOS), and 3-aminopropyl trimethoxy-silane (APS) on the surface of AZ31 magnesium alloy by the sol-gel method and embedded ZnO nanoparticles to form a dense three-dimensional network structure coating on the surface, so as to improve the corrosion resistance of AZ31 magnesium alloy. The experimental results show that the coating can significantly reduce the corrosion current density, improve the electrochemical impedance, and effectively block the intrusion of corrosive medium. The addition of ZnO nanoparticles further enhances the protective properties of the coating, improves the thickness and densification of the coating, and reduces the porosity, so as to effectively inhibit the diffusion of corrosive ions, providing a new method for corrosion protection of absorbable magnesium alloy implants.

From the above article, it can be seen that the traditional silane sol-gel coating has good initial corrosion resistance and biocompatibility due to its hydrophobic Si-O-Si network and easy surface modification. However, due to the fact that covalent bonds in sol-gel matrices are prone to hydrolysis in physiological environments, their long-term durability is limited. To overcome this issue, biological modifications of molecules such as dopamine (DOPA), catechol (CA), and lysine (Lys) were introduced, significantly enhancing the coating density, defect sealing performance, and prolonging the anti-corrosion time. These modified coatings exhibit excellent performance, extending the protection period from several days to over 2 weeks in salt water environments while maintaining environmental friendliness and low cytotoxicity, which is of great significance for clinical applications. In addition, the mixed sol-gel system combined with bioactive ions (Ca, Mg) or nanoparticles (ZnO, SiO_2_) provides multifunctionality by enhancing corrosion resistance and biological activity, supporting its application in orthopedic implants. However, excessive addition of these additives may cause porosity and damage the stability of the coating. Overall, sol-gel coatings, especially those with bioinspired or mixed-enhanced properties, have demonstrated strong clinical potential in biodegradable magnesium implants. However, long-term performance, additive balance, and process optimization remain challenges for wide application.

#### 3.3.2. Layered Coatings

**Figure 9:**
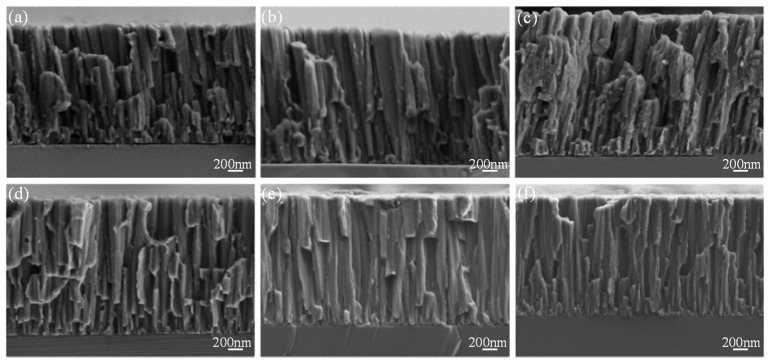
SEM cross section of the coating: (**a**) Mg-0.0Zr; (**b**) Mg-0.4Zr; (**c**) Mg-1.0Zr; (**d**) magnesia-2.0Zr; (**e**) Mg-3.4Zr; (**f**) magnesium −5.0Zr [54].

A laminated coating is a type of coating based on the number of layers applied or the design of the coating structure, primarily by depositing or building up different layers of material layer by layer to achieve a specific function or performance. In order to regulate the bioabsorption properties of magnesium alloys, Benzarti et al. [54] prepared Mg-Zr layered coatings with different contents on the surface of magnesium alloys by DC magnetron sputtering. It was found that doping Zr (1.0–3.4 at.%) caused the coating to form a dense column structure (Figure 9), and the grains were also refined. In addition, the corrosion rate of magnesium alloy decreased from 45 mm/year of pure Mg to 1–12 mm/year. With the increase of Zr content, the corrosion resistance and hardness of the coating also increase, while the surface morphology and free energy also change. The corrosion current density of the MG-3.4Zr coating is only 31 μA/cm^2^, which is 98% lower than that of pure Mg (1946 μA/cm^2^).

Basically, the performance of magnesium alloys as biodegradable orthopedic implants can also be improved by using coatings of different structures on top of each other. Li et al. [55] prepared a multifunctional composite coating on the surface of magnesium alloy by a layer-by-layer deposition technology, which is composed of three layers: The bottom layer is the oxide layer formed by plasma electrolytic oxidation (PEO); the middle layer is the ZnO layer deposited by hydrothermal method; and the outermost layer is the metal–organic framework (MOF, ZIF-8) layer synthesized by the solvothermal method. Compared with the PEO coating, the corrosion current density of the composite coating decreased from 7.798 × 10^−8^ A/cm^2^ to 8.757 × 10^−8^ A/cm^2^, the friction coefficient decreased from 0.7 to 0.2, and the cellular activity increased from 40% to 80%. The coating remained intact after immersion in simulated body fluids for 5 days, demonstrating excellent long-term stability. The experimental data show that the coating significantly improves the corrosion resistance, biocompatibility, and wear resistance of magnesium alloy.

**Figure 10:**
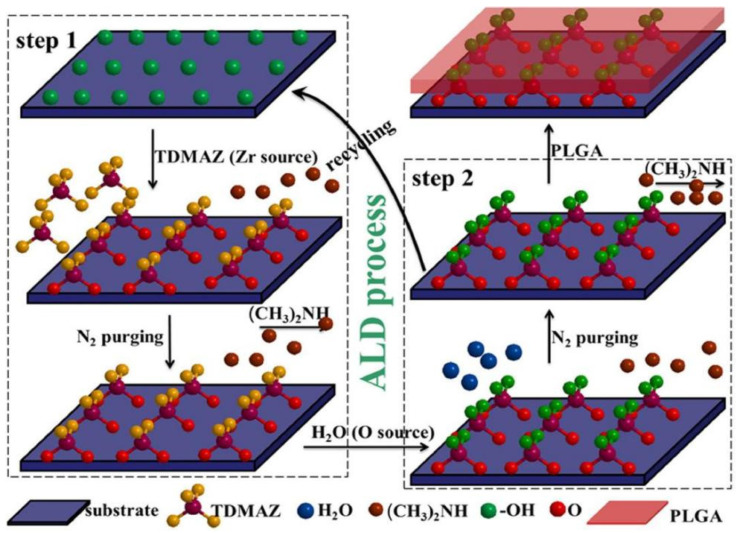
Schematic diagram of formation mechanism of ZrO_2_ coating deposited by ALD and its PLGA overlay [56].

Liu et al. [56] proposed a composite coating technology based on atomic layer deposition (ALD), which significantly improved the corrosion resistance of AZ31 magnesium alloy by successively deposition of ZrO_2_ nano-films (deposition rate 0.117 nm/cycle) and spin coating of polylactic acid–hydroxyacetic acid (PLGA) on the surface of AZ31 magnesium alloy. Experiments show that increasing ALD cycles (25 to 100 times) can increase the thickness of ZrO_2_ coating and reduce the corrosion rate by 2–3 orders of magnitude. PLGA further fills the nanogap and delays electrolyte penetration. However, once the ZrO_2_ membrane is damaged, the local acid and galvanic corrosion caused by PLGA hydrolysis will accelerate the substrate corrosion. The formation mechanism of ZrO_2_ coating deposited by ALD and its PLGA coating is shown in Figure 10. This strategy optimizes coating properties by adjusting ALD process parameters and provides a controllable and corrosion-resistant surface modification scheme for biodegradable magnesium alloy implants.

Nadaraia et al. [57] prepared a new superhydrophobic composite coating on Mg-Mn-Ce magnesium alloy. The coating was formed by plasma electrolytic oxidation on the surface of magnesium alloy to form a ceramic matrix, which was then sequentially coated with super-dispersed polytetrafluoroethylene and sprayed with tetrafluoroethylene oligomer solution. The coating has irregular delamination results, low surface free energy, and a contact angle of 171 ± 2°, showing superhydrophobic properties. At the same time, the corrosion current density is as low as (1.7 ± 0.1) × 10^−9^ A cm^−2^, and the wear rate is as low as (9.8 ± 0.5) × 10^−6^ mm^3^ N^−1^ m^−1^, which can effectively reduce the corrosion and wear of magnesium alloy, and can maintain stability in high temperature, low temperature, and corrosive environments. After 40 days of a salt spray test, the coating still has good protection performance.

Coatings containing nanomaterials can provide nanoscale surfaces and controllable roughness and hydrophilicity for substrates and promote cell adhesion and proliferation. They also exert antibacterial activity and enhance osteoblast differentiation and endothelial migration to accelerate tissue repair. In addition, compared with traditional large-particle coatings, nanocoatings have high-density grain boundaries, interphase boundaries, and dislocations, thereby enhancing corrosion resistance [31]. Inspired by fish scales, Li et al. [111] developed a biomimetic nanocoating for infectious bone defects. This coating, composed of black phosphorus (BP) and graphene oxide (GO), is assembled onto a degradable magnesium alloy implant via a pulsed electric field. The BP/GO coating demonstrated excellent photothermal antibacterial performance in vitro, effectively inhibiting the growth of Staphylococcus aureus and Escherichia coli, with an antibacterial rate exceeding 99%. In in vivo experiments, the BP/GO coating significantly promoted vascularized bone regeneration, and the bone volume relative to tissue volume (BV/TV) increased by 3.25 times at 8 weeks compared with the control group. This coating promotes the migration of vascular endothelial cells by regulating microtubule deacetylation, creating a favorable microenvironment for bone repair. Dai et al. [112] prepared a biomimetic superhydrophobic composite coating on the surface of magnesium alloy through femtosecond pulsed laser processing, in situ growth of Mg-Al layered double hydroxides (LDHs), and modification with low-surface-energy materials. The coating features a dual-scale micro–nano structure and exhibits excellent corrosion resistance, with the corrosion current density reduced by approximately five orders of magnitude compared to bare magnesium alloy. It also possesses superhydrophobicity (contact angle of 154.60°), anti-icing properties (ice formation time prolonged by 250% at −40 °C), self-cleaning, and anti-fouling capabilities and maintains its superhydrophobicity after multiple cycles of sandpaper abrasion and tape-peeling tests.

#### 3.3.3. Ionic Liquid Conversion Coatings

An ionic liquid conversion coating (ILCc) is a protective coating formed by the chemical reaction of an ionic liquid with a metal surface. Ionic liquid (IL) refers to a liquid composed entirely of ions. The main difference between IL and molten salt is the low melting point, because IL is already liquid at or near room temperature (melting point < 100 °C). Ionic liquids have low volatility, high thermal stability, good electrical conductivity, and unique chemical properties and are currently mainly used in the fields of electrochemistry, catalysis, and chemical synthesis [113]. Howlett et al. [58] studied the corrosion protection properties of magnesium alloy AZ91D by using trihexyl (tetradecyl) phosphorus bis (trifluoromethanesulfonyl) amide ionic liquid conversion coating. After activation and conditioning pretreatment, AZ91D was immersed in ionic liquid to form the conversion coating. The electrochemical test and surface analysis results showed that the corrosion rate of AZ91D alloy after A + C pretreatment and ionic liquid coating was significantly reduced, the corrosion potential was corrected, and the corrosion resistance was improved. The surface morphology observation showed that the pretreatment changed the microstructure and electrochemical reactivity of the alloy surface, and the ionic liquid coating formed a more uniform and dense protective film on the A + C pretreatment surface, which effectively hindered the anodic dissolution of magnesium. The experimental results show that the ionic liquid conversion coating provides effective corrosion protection for magnesium alloy AZ91D, especially when combined with A + C pretreatment, which provides a new option for chromate replacement coating. However, the coating formation time is longer, and further studies on the coating formation mechanism, influencing factors, and optimization conditions are needed to improve the coating performance and practicability.

In addition, Elsentriecy et al. [114] studied the conversion coating formed by aprotic ammonium phosphate ionic liquid on the surface of magnesium alloy, and discussed the effects of pretreatment and process temperature on corrosion protection properties. The results showed that treatment at 300 °C was more effective than at room temperature, which was attributed to the fact that the ionic liquid reacted with the surface of magnesium alloy to form a protective film containing metal phosphate and oxide after thermal decomposition. Phosphoric acid pickling combined with high-temperature ionic liquid treatment showed a synergistic effect, and the conversion coating formed was mainly composed of metal phosphate, metal oxide, and organic compounds; it had good anti-corrosion properties and was expected to replace the toxic chromate. Subsequently, Elsentriecy et al. [115] used proton ammonium phosphate ionic liquid to treat AZ31B magnesium alloy at 300 °C, forming a 70–80 nm thick double-layer conversion coating, which was mainly composed of metal oxides, metal phosphates, and carbon compounds, significantly improving the corrosion resistance of magnesium alloy. Electrochemical tests showed that the treated samples had the lowest corrosion current density and the strongest pitting resistance. It can be seen that thermal stress promotes the decomposition of the ionic liquid, and the decomposition products react with the alloy surface to form a dense conversion coating, which effectively improves the corrosion resistance of magnesium alloy.

**Figure 11:**
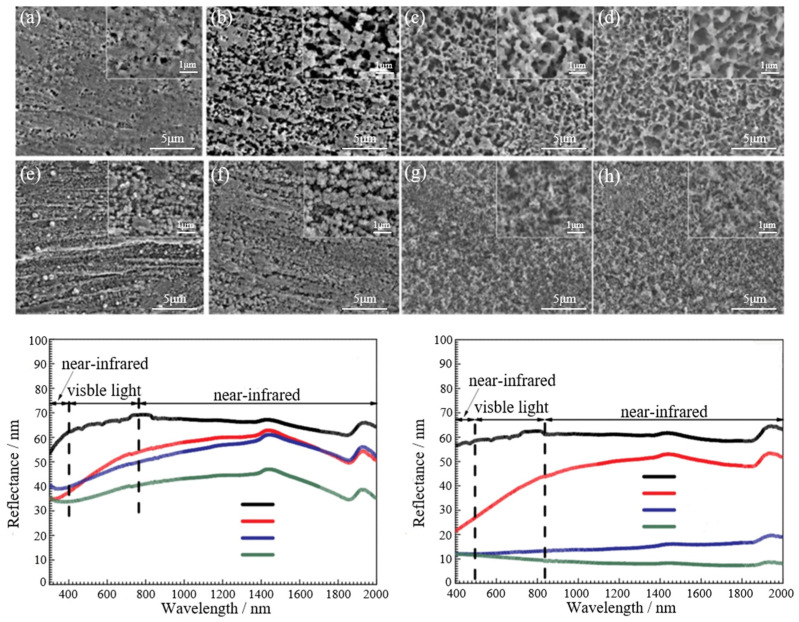
SEM morphologies of Mg/AZ31B substrate at different thermal corrosion times at 160 °C: (**a**) Mg-5; (**b**) Mg-15; (**c**) Mg-30; (**d**) Mg-60; (**e**) AZ-5; (**f**) AZ-15; (**g**) AZ-30; (**h**) AZ-60; Reflection spectra of pure Mg (**i**) and AZ31B (**j**) matrices after thermal corrosion [117].

Deep eutectic solvent (DES) is a new type of ionic liquid. Compared with traditional IL, DES has several advantages; it has lower toxicity and is easier to obtain and prepare [116]. Guo et al. [117] proposed a method to improve the corrosion resistance of magnesium alloy by pretreating with deep eutectic solvent (DES) and combining with hydrophobic epoxy resin coating. Through experiments, it was found that DES pretreatment formed a porous transformation film mainly composed of MgH_2_, MgO, and MgCO_3_ on the surface of magnesium alloy. This porous structure provides more attachment points for the subsequent epoxy resin coating (Figure 11), and the dual-layer coating structure pretreated by DES greatly improves the corrosion resistance of magnesium alloy.

Zhang et al. [118] anodized AZ31B magnesium alloy with a choline chloride–ethylene glycol deep eutectic solvent (DES) to prepare a MgCO_3_ conversion film. The experiments showed that the CF-50 membrane formed at a current density of 50mA cm^−2^ was a nanorod array structure, and the corrosion current density (i_corr) decreased from 988 μA cm^−2^ to 27.7 μA cm^−2^ of the bare alloy. After further modification by SHS-50 and SLIPS 50, the i_corr decreased to 0.711 μA cm^−2^ and 0.263 μA cm^−2^, respectively. The contact angles reached 158° and 117°. In the immersion experiment, SLIPS-50 showed only a slight corrosion point after 24 h in 3.5% NaCl solution, which was significantly better than the severe corrosion of bare alloy. This process provides a new strategy for effective corrosion protection of magnesium alloys by regulating current density and surface modification. At present, EDS-based coatings are thin and porous, which can only prevent corrosion for a short period of time, and most coatings containing organic solvents are not environmentally friendly. Therefore, in order to improve the corrosion resistance of DES-based conversion films, organic coatings are necessary. At the same time, more efficient coatings on degradable Mg alloys are expected to be prepared by applying different external fields at the IL/substrate interface [119].

#### 3.3.4. Hybrid Coatings of Bioactive Molecules

Magnesium alloy–bioactive molecule hybrid coating is a technology that enhances the corrosion resistance, biocompatibility, and bioactivity of magnesium alloys by constructing composite coatings on their surfaces. This type of coating usually combines inorganic, organic, or biomolecular components to form a hybrid structure to meet the requirements of medical implant materials (such as bone nails, vascular stents, etc.).

**Figure 12:**
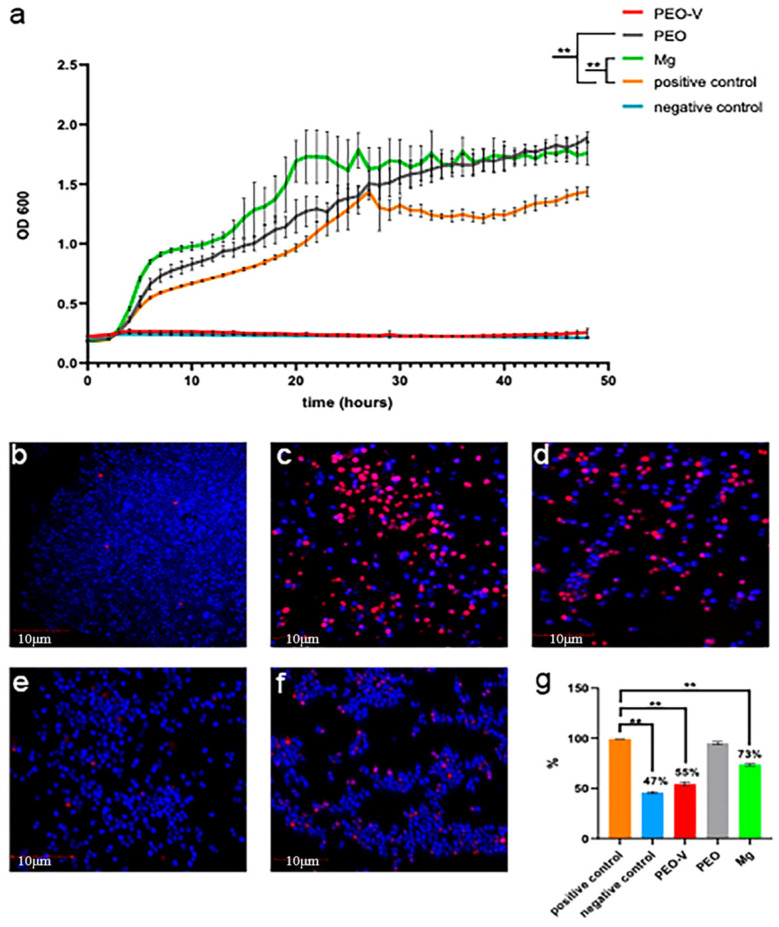
Growth curves of Staphylococcus aureus in the presence of the studied materials for 48 h (**a**). Relevant images from live/dead assay of S. aureus culture under the influence of the studied materials, including positive and negative controls: positive control (**b**), negative control (**c**), PEO-V (**d**), PEO (**e**), Mg (**f**). Red fluorescence (propidium iodide) is for dead cells; blue fluorescence (Hoechst 33342) is for live cells. Viability of S. aureus in the presence of the studied materials after cultivation for 24 h (**g**). ** *p* < 0.01 [121].

Vancomycin is a glycopeptide antibiotic mainly used to treat severe infections caused by drug-resistant Gram-positive bacteria. Its mechanism of action is to interfere with the synthesis of bacterial cell walls, combine with D-Ala-D-Ala, prevent the synthesis of peptidoglycan, and cause the death of bacteria. It is effective against Staphylococcus aureus, Streptococcus, Enterococcus, etc., having an especially good therapeutic effect on methicillin-resistant Staphylococcus aureus (MRSA), and thus is widely used in the medical field and other areas [120]. Nadaraia et al. [121] formed a coating containing hydroxyapatite on the surface of MG-MN-CE alloy through plasma electrolytic oxidation (PEO) technology and loaded vancomycin to enhance the antibacterial performance. Experiments show that this coating significantly enhances the corrosion resistance of magnesium alloys, effectively inhibits the growth of Staphylococcus aureus, and demonstrates excellent biocompatibility and low toxicity in both in vitro and in vivo experiments (Figure 12), with the potential to lead a new generation of bioabsorbable implant materials.

Bone morphogenetic protein-2 (BMP-2) mainly functions in the research by promoting osteocyte differentiation, stimulating new bone formation, improving the biocompatibility of implants, and controlling the degradation rate of magnesium alloys [122]. This pleiotropy makes BMP-2 a key factor in enhancing the performance of magnesium alloys as orthopedic and dental implant materials. Kim et al. [59] studied the control of the bioabsorption rate and the improvement of the biocompatibility of magnesium alloys by combining different concentrations of BMP-2 on their surfaces through layer assembly (LBL) technology. The carrier layer was formed through micro-arc oxidation and hydrothermal treatment, and then BMP-2s of different concentrations were fixed. The experiment evaluated the surface characteristics, electrochemical corrosion behavior, cytotoxicity, and bone formation ability of these coatings in vivo. The results show that the coating can significantly improve the corrosion resistance and biological activity of magnesium alloys. Among them, the BMP-2 concentration group of 50 ng/mL has the best effect in promoting bone formation and stabilizing bone growth.

### 3.4. Metal-Based Coatings

Metal-based coatings, particularly metal hydroxide and metal oxide coatings, are essential for improving the corrosion resistance, mechanical properties, and biocompatibility of magnesium alloys, which are prone to rapid degradation. Magnesium hydroxide coatings, such as Mg(OH)_2_, offer advantages like biocompatibility and self-healing properties, while metal oxide coatings, including MgO, Al_2_O_3_, and TiO_2_, enhance both corrosion and wear resistance. These coatings are widely used in applications like medical implants, where prolonged material performance is crucial.

#### 3.4.1. Metal Hydroxide Coatings

The corrosion products of magnesium in solution are usually loose Mg(OH)_2_, which can easily react with chloride ions in the environment, thereby accelerating the corrosion rate of magnesium alloys. Moreover, since magnesium hydroxide is also a degradation product in the human body, it has biocompatibility [123]. Therefore, some scholars have attempted to prepare a dense magnesium hydroxide coating in order to improve the corrosion resistance of the magnesium alloy surface. Xie et al. [60] studied an environmentally friendly and simple Mg(OH)_2_ film for the nickel-free electroplating (NIP) coating of magnesium alloys, thereby enhancing the inhibition of galvanic corrosion. The research successfully achieved the deposition of Ni-P alloy coatings by preparing Mg(OH)_2_ conversion films on the surfaces of magnesium alloys and activating them with AgNO_3_. The experimental results show that this coating exhibits excellent resistance to galvanic corrosion in neutral salt spray tests and NaCl-solution immersion. Compared with the traditional phosphate pretreatment, it has a longer corrosion protection life. Wang et al. [124] successfully prepared Mg(OH)_2_ layers with a thickness of approximately 25.5 ± 1.2 microns on the surface of magnesium alloys by oxidizing them in steam at 200 °C for 4 h. Tribological tests show that this coating significantly reduces the friction coefficient and wear rate of magnesium alloys. Corrosion tests show that the coating significantly enhances the corrosion resistance of magnesium alloys in a 3.5% NaCl solution. This technology provides an environmentally friendly and effective method for improving the performance of magnesium alloys. Wang et al. [125] prepared a Mg(OH)_2_@MXene bilayer coating on AZ31B magnesium alloy by the one-step hydrothermal method. Ti_3_C_2_Tx MXene can not only promote the growth of Mg(OH)_2_ but also binds stably with it. The experimental results show that this coating significantly improves the anti-corrosion performance of magnesium alloys, providing a new method for preparing high-performance magnesium alloy coatings. In addition, some scholars have introduced functional metal ions to replace part of Mg^2+^, forming a composite hydroxide layer that has both corrosion protection and biological functions. For instance, Wu et al. [126] prepared a Mg(OH)_2_-SiO_2_-Al(OH)_3_ composite coating on the surface of AZ91D magnesium alloy by a one-step in situ hydrothermal method. This coating is composed of an aluminum-rich Mg(OH)_2_ base layer and SiO_2_-Al(OH)_3_ microsphere particles, significantly improving the corrosion resistance of magnesium alloys. The coating is firmly bonded to the substrate, and the silicate generated during the hydrothermal process further enhances the stability of the coating. Electrochemical experiments show that this composite coating can significantly reduce the corrosion current density and enhance the corrosion resistance of magnesium alloys.

Recently, layered dihydroxides have gradually come into view as a new type of surface protection material. Due to their advantages, such as good corrosion resistance, self-healing ability, environmental friendliness, multi-functionality, and easy preparation, they are widely used in the surface treatment of magnesium alloys [127]. Wang et al. [61] investigated the effect of citric acid (CA) pretreatment on the formation and corrosion resistance of Mg-Al layered double hydroxide vapor coating on the surface of AZ80 magnesium alloy. The results show that CA pretreatment significantly enhances the surface activity of the alloy, increases the surface area fractions of Mg_17_Al_12_ and Al_8_Mn_5_ phases, and promotes the growth of subsequent LDH coatings. After 30 s of CA pretreatment, the coating became denser, and its thickness increased. The corrosion current density was three orders of magnitude lower than that of the uncoated alloy, demonstrating excellent corrosion resistance. Li et al. [128] incorporated LDH nano-containers into the plasma electrolytic oxidation coating of AZ91 magnesium alloy in situ, thereby enhancing the corrosion resistance of the coating. By adding Zn-Al LDH nano-containers to the PEO electrolyte and loading maleic acid as a corrosion inhibitor, it was found that the thickness and density of the coating increased. Electrochemical tests show that the addition of LDH nano-containers and inhibitors significantly enhances the corrosion resistance of the coating, enabling it to exhibit more stable and excellent corrosion protection capabilities in NaCl solution.

The above studies review various coating methods for improving the corrosion resistance of magnesium alloys, each offering unique added value and potential clinical applications. Dense Mg(OH)_2_ coatings, due to their biocompatibility and environmental friendliness, are effective in suppressing galvanic corrosion and enhancing wear resistance; however, their limited functionality may constrain their use in complex clinical environments. Composite coatings incorporating MXene or functional metal ions further improve structural stability and multifunctionality, offering stronger adhesion and enhanced corrosion resistance, showing promise for high-performance biomedical applications. Layered double hydroxide (LDH) coatings have gained increasing attention for their excellent corrosion resistance, self-healing ability, and eco-friendly nature. When combined with citric acid pretreatment, drug-loaded nano-containers, and biomimetic structures, LDH coatings demonstrate significantly reduced corrosion current density, along with additional functions such as anti-icing, anti-fouling, and mechanical durability, making them highly suitable for clinical use. However, the complex preparation processes and sensitivity to treatment conditions may limit the large-scale clinical application of LDH coatings. Overall, composite and multifunctional coatings represent the future direction of surface treatments for magnesium alloys, though further improvements are needed in fabrication, long-term stability, and biosafety.

#### 3.4.2. Metal Oxide Coatings

Magnesium alloy metal oxide coatings mainly include magnesium oxide coatings, aluminum oxide coatings, and zirconia and titanium oxide coatings. Among them, the magnesium oxide coating is mainly achieved by applying a high voltage to the surface of magnesium alloy through MAO technology, triggering a tiny spark discharge, which melts the surface oxide and forms a dense oxide layer. This process involves complex physicochemical reactions, including the dissolution of oxides, the release of oxygen, and the local melting and recrystallization of the oxide layer. The main chemical formulas involved are [129]

(5) $$\begin{array}{c} Mg\to {Mg}^{2+}+2e^{-} \end{array}$$

(6) $$\begin{array}{c} {4OH}^{-}\to O_{2}↑+{2H}_{2}O+4e^{+} \end{array}$$

(7) $$\begin{array}{c} {2H}_{2}O\to {2H}_{2}↑+O_{2}↑ \end{array}$$

(8) $$\begin{array}{c} {Mg}^{2+}+{2OH}^{-}\to {Mg\left(OH\right)}_{2}↓ \end{array}$$

(9) $$\begin{array}{c} {Mg\left(OH\right)}_{2}\to MgO↓+{2H}_{2}O \end{array}$$

(10) $$\begin{array}{c} 2Mg+O_{2}\to MgO↓ \end{array}$$

The MAO coating has a porous structure, which can enhance the mechanical interlocking effect, but the pores may also become channels for corrosion. By adjusting parameters such as electrolyte composition, current density, and treatment time, the thickness, porosity, and uniformity of the coating can be controlled, thereby enhancing its corrosion resistance. Zhang et al. [130] discussed the application of magnesium oxide coating and plasma electrolytic oxidation (PEO) technology for AZ61 magnesium alloy. By adjusting the electrolyte composition and voltage, the ordered discharge mode (OD) was achieved, optimizing the formation process of the PEO coating. Research shows that when the mass ratio of magnesium fluoride to magnesium oxide is 1.3 and the voltage is 130 V, the electron avalanche can be effectively limited, forming a thicker inner dense layer and a denser outer layer, thereby significantly improving the corrosion resistance and wear resistance of the coating. Dong et al. [131] prepared ultra-wear-resistant coatings on the surface of magnesium alloys through PEO and discontinuous deposition (DCS) techniques. PEO treatment can improve the corrosion resistance of magnesium alloys, but the approximately 14-micron-thick and rough protective layer it generates has poor wear resistance. Researchers deposited a 1.3-micron-thick polytetrafluoroethylene polymer layer on the PEO layer in a discontinuous manner through selective spraying, increasing the wear resistance by approximately 5500 times. DCS forms nano-cracks, splitting the coating into multiple layers of nano-blocks. During the friction process, the movement of the nano-blocks generates rolling friction and nano-lubrication effects, significantly enhancing wear resistance.

Alumina coating in medical magnesium alloys can enhance corrosion resistance and biocompatibility, improve mechanical properties and antibacterial effects, and play an important role in prolonging the service life of implants and promoting postoperative recovery. Jin et al. [132] studied the effect of AlO_2_^2−^ on the PEO coating of AZ61 magnesium alloy by adding different concentrations of NaAlO_2_ to the KF-KOH electrolyte. The results show that the addition of AlO_2_^2−^ leads to the appearance of a new-phase MgAl_2_O_4_ in the coating. With the increase of its concentration, the porosity of the coating decreases, the surface morphology improves, and the corrosion resistance and wear resistance are enhanced. When the concentration of NaAlO_2_ is 15 g/L, the coating performance is the best, the porosity is the lowest (3.23%), and the corrosion resistance and wear resistance are optimal. Studies show that the addition of AlO_2_^2−^ is an effective method to improve the coating quality of magnesium alloys.

Medical magnesium alloys are prone to corrosion in the complex physiological environment of the human body. The titanium dioxide coating can act as a barrier to slow down the corrosion rate of magnesium alloys. Moreover, the titanium oxide coating itself has good biocompatibility and can enable human cells to adhere, proliferate, and differentiate well on its surface [133]. Mashtalyar et al. [134] formed a coating containing titanium dioxide (TiO_2_) nanoparticles on the surface of magnesium alloys through PEO. These coatings improve the corrosion resistance and mechanical properties of magnesium alloys, reduce the corrosion current density, and increase the hardness. Confocal micro-Raman spectroscopy revealed that there were anatase and rutile phases in the PEO coating, and the addition of TiO_2_ nanoparticles enhanced the photocatalytic performance of the coating, increasing the decomposition rates of methyl orange and methylene blue by 1.6 times and 1.8 times, respectively. Research indicates that the addition of TiO_2_ nanoparticles is an effective method to enhance the performance of magnesium alloy coatings. Han et al. [135] significantly improved the corrosion resistance and biocompatibility of the TiO_2_ coating by using atomic layer deposition technology on the micro-arc oxidation coating of AZ31 magnesium alloy. The research found that the TiO_2_ layer effectively sealed the micropores and microcracks of the MAO coating, enhanced the corrosion resistance, and maintained the surface morphology. In vitro cell experiments have shown that the TiO_2_/MAO composite coating is more biocompatible than the substrate and MAO coating, providing a new idea for applications in the field of bioremediation.

Magnesium alloy surface coating technology has significant advantages in enhancing corrosion resistance, wear resistance, and biocompatibility. Magnesium oxide coatings are formed through MAO technology and have excellent corrosion resistance and wear resistance, but their porous structure may lead to the formation of corrosion channels. Alumina coatings exhibit excellent corrosion resistance and biocompatibility on medical magnesium alloys, but precise control of electrolyte composition and processing parameters is required. Titanium oxide coatings not only have good biocompatibility but can also significantly improve photocatalytic performance by adding TiO_2_ nanoparticles. Each coating technology has its unique added value and limitations. Choosing the appropriate coating technology requires comprehensive consideration based on specific application requirements and environmental conditions. Future research directions should focus on further optimizing the coating formation process, enhancing the uniformity and performance of the coating to meet the demands of a wider range of clinical applications.

## 4. Potential Challenges of Surface Modification of Medical Magnesium Alloys

Surface modification technology can provide magnesium-based materials with excellent mechanical properties, good biocompatibility, and degradability, and it has broad applications in the field of biomedical-related materials. However, the corrosion degradation mechanism and biocompatibility of magnesium-based materials are still facing certain challenges. Rare earth element-doped magnesium matrix composites are one example. These materials have low-density and high-strength mechanical properties, showing an important application value in the field of orthopedic instruments, but their degradation kinetics control is still facing challenges. The ionic dissolution and biocompatibility of some alloying elements will adversely affect the bone healing process, which is a big challenge for the medical application of magnesium-based materials. Large-sized magnesium alloy implants face significant clinical challenges due to hydrogen evolution at elevated resorption rates, while stress gradients inherent in macroscopic components accelerate localized corrosion rates, collectively compounding the difficulties for in vivo implantation. Therefore, the construction of a corrosion prevention mechanism of multi-scale materials and the improvement of the biocompatibility of materials can better provide theoretical support for the optimal design of a new generation of degradable magnesium-based implants.

In recent years, the research on anti-corrosion and biocompatibility of magnesium alloy surface coating technology has made great progress, and some technical systems have achieved clinical transformation [136]. However, although the biggest limitation of magnesium-based materials in vivo is their degradation rate and biocompatibility, other interface characteristics, such as adhesion/fatigue, interphase diffusion behavior, and mechanical properties, are important factors in the quality and stability of coatings, to improve the adhesion and fatigue strength of magnesium alloys in the organismal environment [137]. It is worth noting that although the relevant literature has discussed the degradation rate, porosity, and conventional evaluation indicators of the non-toxicity of corrosion products of Mg-based alloys, the adhesion test for evaluating the stability of polymer coatings is rarely reported. In particular, there is a lack of a unified thermodynamic–kinetic coupling analysis model. In addition, the synergistic mechanism between the solution osmotic pressure, crystallinity distribution, phase transition temperature, and degradation microenvironment is essential for a comprehensive understanding of coating formation in magnesium-based alloys. Research into the above problems will play an important role in the commercial application of Mg-based materials.

## 5. Summary

In summary, excellent mechanical properties and good biocompatibility and degradability make magnesium alloy a promising biomedical material. However, there are still great challenges in the development of advanced coatings for biomedical magnesium alloys. In order to harness the development potential of magnesium alloys in vivo, further research can be carried out in the following aspects:

1. The interaction mechanism between the magnesium alloy matrix and the modified layer and the modified layer and the biological interface should be deeply studied and the interface characteristics of the coating fully considered, such as the influence of adhesion force, interphase diffusion, and mechanical properties, to provide a theoretical basis for the preparation of the surface coating.
2. Develop multifunctional composite coatings according to the requirements of high corrosion resistance, self-degradation, drug resistance, and biosafety of clinical biomedical implant materials. Prepare the bottom layer with a good bond to the substrate, introduce the polymer coating onto the prepared coating by chemical combination, and then conjugate the corrosion inhibitor molecules to the hybrid coating. It can effectively avoid the shortcomings of limited interface bonding strength between different coatings in the composite coating and prepare a multifunctional and integrated coating with controlled release, self-healing, and good biocompatibility.
3. Nanoscale characterization at the interface is still lacking, especially in situ deposition/growth, and cannot yet provide strong evidence for the interaction between degradation products and surrounding tissues. New characterization systems can be established by combining advanced sensing technology and big data analysis.
4. Optimizing material design to regulate alloy phases and nanostructures, thereby constructing functionally graded magnesium alloy coating systems through multi-scale collaborative design, will alleviate the constraints imposed by hydrogen evolution at elevated resorption rates in large-sized magnesium alloy implants.

## Figures and Tables

**Table 1:** Various coatings applied to Mg alloys.

Substrate	Materials	Coating	Results	Ref.
AZ31B	MAO/FSL	HAp	It has the most reasonable degradation rate and exhibits excellent biocompatibility and antibacterial performance.	[32]
AZ91	MAO	MAO-LBL	The mechanical interlocking and adhesion of the coating have been enhanced, further preventing the penetration of corrosive media.	[33]
AZ31B	MAO	MAO/DLC	After PDMS penetrates the pores of the coating, it can enhance the density, adhesion, wear resistance, and corrosion resistance of the coating.	[34]
AZ91D	Chemical conversion	Phosphate	The presence of the strong oxidant KMnO_4_ forms an extremely thin passivation film on the protruding β phase, which inhibits the formation of phosphate crystals and prevents the shielding of conductive points.	[35]
AZ31	Soaking	MgF_2_	The coating significantly reduces the corrosion rate of AZ31 magnesium alloy, but the defect still leads to local corrosion.	[36]
AZ31B	Hydrothermal	Mg_HT_/TA-APTES/PDMS	The corrosion rate of magnesium alloys is significantly reduced, and they possess excellent chemical stability and mechanical durability.	[37]
Pure Mg	Biomimetic deposition method	HAp	The corrosion behavior of the coating is influenced by surface pretreatment and deposition time. The coating deposited 6 h after heat treatment has higher corrosion resistance.	[38]
Mg alloy	Hydrothermal	PPA-HAp	Compared with traditional HA coatings, PPA-HA coatings have higher adhesion and better hardness and elastic moduli and have demonstrated excellent long-term corrosion resistance in in vitro experiments.	[39]
WE43	Anodic oxidation	Ca-P	The HA coating is converted from DCPD through alkaline treatment and exhibits excellent microstructure, adhesion strength, and corrosion resistance.	[40]
Pure Mg	Soaking	CaP	Both coatings significantly enhance the corrosion resistance of magnesium substrates. Among them, the DCPD coating demonstrates superior corrosion resistance due to its low porosity, thickness, and good coverage.	[41]
AZ31, AZ91	Plasma spraying	HAp	The experimental results show that the HAp coating significantly reduces the corrosion rate of the alloy in the simulated fluid, from 1.2 mm/year without coating to 0.4 mm/year.	[42]
AZ31	Plasma spraying	HAp/Sr	Electrochemical tests show that the corrosion current density of the HA + 12%Sr coating is the lowest, demonstrating the best corrosion resistance.	[43]
AZ31	Hydrothermal and dipping methods	PCL/HAp	PCL fills the pores of HA crystals to form a dense structure, which enhances the bonding strength of the coating and reduces the electrochemical corrosion rate to 6.9 mm/year.	[44]
ZM21	Combining double emulsification and phase separation techniques	PA-PCL	The increase in pH value and the increase in Mg2 concentration respectively increase the PA release by 2.5 times and 3.1 times, further enhancing the self-repairing ability of the coating.	[45]
AZ31, WE43, AZ91	Chemical vapor deposition	Parylene C	The results show that the organic coating significantly enhances the corrosion resistance of the alloy; Parylene C can effectively delay the degradation of magnesium alloys while maintaining mechanical adaptability.	[46]
AZ31	Coprecipitation method	Chitosan/LDH	This intelligent coating can trigger the release of corrosion inhibitors at different pH values, and the coating containing gallic acid ions significantly improves the corrosion resistance of magnesium alloys.	[47]
AZ31	PEO	HA/CMC	It was found through experiments that the HA coating significantly improves the initial corrosion resistance and has a self-healing ability, which could quickly self-repair the damage in the scratch test.	[48]
AZ31B	Anodic oxidation	Sol-Gel	The results show that the coating can significantly improve the corrosion resistance of magnesium alloy at both 110 °C and 160 °C curing temperatures, and the protection efficiency of the 110 °C curing coating reaches 98.6% after 72 h immersion.	[49]
AZ31B	Sol-Gel	GPTMS/TEOS	The coating is uniform and dense and bonds well with the substrate. Moreover, this coating can reduce the corrosion current density by three orders of magnitude, demonstrating excellent anti-corrosion performance.	[50]
AZ31	Sol-Gel	DOPA-modified	The experimental results show that the DOPA-modified coating can provide long-term corrosion protection for more than 14 days in 0.1 M NaCl solution, while the unmodified coating could only maintain 2–3 days.	[51]
AZ31	CA/Ly polymerization	Sol-Gel	Characterization confirms that CA/Lys is environmentally friendly by bonding to silane networks via hydrogen and chemical bonding (CA toxicity is lower than chromate).	[52]
Elektron 21	Sol-Gel	TEOS–GPTMS	The study confirms that the TEOS–GPTMS hybrid system is an effective method to construct dual-function coatings, but the salt content and aging time need to be optimized to balance corrosion protection and biological activity.	[53]
Mg	DCMS	Mg-Zr	With the increase of Zr content, the corrosion resistance and hardness of the coating also increase, while the surface morphology and free energy also change.	[54]
Mg-1Zn-1Gd	LBL	PEO-ZnO@MOF	According to the in vitro biocompatibility assays, the PEO-ZnO@MOF sample shows uniquely outstanding properties of cell proliferation, cell adhesion, and cell viability, compared with those of the PEO, PEO–ZnO, and bare ZG11 samples.	[55]
AZ31	ALD	ZrO_2_/PLGA	PLGA further fills the nanogap and delays electrolyte penetration. However, once the ZrO_2_ membrane is damaged, the local acid and galvanic corrosion caused by PLGA hydrolysis will accelerate the substrate corrosion.	[56]
Mg-Mn-Ce	PEO	MCC	The coating has irregular delamination results, low-surface free energy, and a contact Angle of 171 ± 2°, showing superhydrophobic properties.	[57]
AZ91D	ILC	[P_6,6,6,14_][NTf_2_]	The experimental results show that the ionic liquid conversion coating provides effective corrosion protection for magnesium alloy AZ91D, especially when combined with A + C pretreatment, which provides a new way for chromate replacement coating.	[58]
AZ31B	LBL	BMP-2	The BMP-2 concentration group of 50 ng/mL has the best effect in promoting bone formation and stabilizing bone growth.	[59]
AZ31	Electroless	Ni-P	The experimental results show that this coating exhibits excellent resistance to galvanic corrosion in neutral salt spray tests and NaCl solution immersion.	[60]
AZ80	Steam method	LDH	The addition of LDH nano-containers and inhibitors significantly enhances the corrosion resistance of the coating, enabling it to exhibit more stable and excellent corrosion protection capabilities in NaCl solution.	[61]

MAO = Micro-arc oxidation, Hap = Hydroxyapatite, LBL = Self-assembly layer by layer, DLC = Diamond-like carbon, PDMS = Polydimethylsiloxane, TA = Tannic acid, APTES = Amino silane, PCL = Polycaprolactone, LDH = Layered double hydroxide, PEO = Plasma electrolytic oxidation, HA = Hyaluronic acid, CMC = Carboxymethyl cellulose, GPTMS = 3-Epoxy-propoxy-trimethoxy-silane, DOPA= Levodopa, TEOS = Tetraethoxy-silane, CA = Catechol, Ly = Catechol, DCMS = DC magnetron sputtering, MOF = Metal organic framework, ALD = Atomic layer deposition, PLGA = Polylactic acid–glycolic acid, MCC = Modified composite coatings, ILC = Ionic liquid conversion, [P_6,6,6,14_][NTf_2_] = The performance of a trihexyl(tetradecyl)phosphonium bis(trifluoromethanesulfonyl)amide, BMP-2 = Bone morphogenetic protein-2.

## Data Availability

No new data were created or analyzed in this study. Data sharing is not applicable to this article.
